# QTL Mapping and Candidate Gene Analysis of Telomere Length Control Factors in Maize (*Zea mays* L.)

**DOI:** 10.1534/g3.111.000703

**Published:** 2011-11-01

**Authors:** Amber N. Brown, Nick Lauter, Daniel L. Vera, Karen A. McLaughlin-Large, Tace M. Steele, Natalie C. Fredette, Hank W. Bass

**Affiliations:** * Department of Biological Science, Florida State University, Tallahassee, Florida 32306-4295; † United States Department of Agriculture—Agricultural Research Service Corn Insects and Crop Genetics Research Unit, Iowa State University, Ames, Iowa 50011-1020; ‡ Department of Plant Pathology and Microbiology, Iowa State University, Ames, Iowa 50011-1020

**Keywords:** IBM, TRF, plant, telomerase, B73

## Abstract

Telomere length is a quantitative trait important for many cellular functions. Failure to regulate telomere length contributes to genomic instability, cellular senescence, cancer, and apoptosis in humans, but the functional significance of telomere regulation in plants is much less well understood. To gain a better understanding of telomere biology in plants, we used quantitative trait locus (QTL) mapping to identify genetic elements that control telomere length variation in maize (*Zea mays* L.). For this purpose, we measured the median and mean telomere lengths from 178 recombinant inbred lines of the IBM mapping population and found multiple regions that collectively accounted for 33–38% of the variation in telomere length. Two-way analysis of variance revealed interaction between the quantitative trait loci at genetic bin positions 2.09 and 5.04. Candidate genes within these and other significant QTL intervals, along with select genes known *a priori* to regulate telomere length, were tested for correlations between expression levels and telomere length in the IBM population and diverse inbred lines by quantitative real-time PCR. A slight but significant positive correlation between expression levels and telomere length was observed for many of the candidate genes, but *Ibp2* was a notable exception, showing instead a negative correlation. A *rad51-like* protein (TEL-MD_5.04) was strongly supported as a candidate gene by several lines of evidence. Our results highlight the value of QTL mapping plus candidate gene expression analysis in a genetically diverse model system for telomere research.

The ends of linear eukaryotic chromosomes are called telomeres, a term coined by Müller (1938). As early as 1919, chromosome ends were recognized as displaying unique behavior during the prophase of meiosis I (Digby 1919). Subsequently, telomeres were found to have distinct roles in stabilizing or capping the ends of eukaryotic chromosomes (McClintock 1941) and in solving the end-replication problem (Olovnikov 1971). More recently, research has revealed widespread conservation of telomere structures and functions across diverse species of plants, animals, protists, and fungi (Blackburn *et al.* 2006). Among the great biological discoveries in the twentieth century was the finding that telomerase regulation of telomere length plays an important role in cell aging and proliferative capacity in human cells (Bodnar *et al.* 1998). Given the variety of telomere functions, the catastrophic genomic effects and organismal consequences of telomere misregulation are not surprising. For example, many forms of cancer are associated with telomere defects, disruption of telomere-capping functions can be lethal (reviewed by Martinez and Blasco 2011), and telomere malfunctions in meiosis can lead to sterility or aneuploidy (McClintock 1941; Hackett *et al.* 2001; Riha *et al.* 2001; Bass *et al.* 2003).

Telomeres are composed of short tandem repeats of DNA, typically in the range of 5–7 bp per repeat, associated with specialized protein complexes (Martinez and Blasco 2011). Telomere-repeat DNA, first identified in the protist Tetrahymena, was found to be a hexameric repeating sequence, CCCCAA/GGGGTT (Blackburn and Gall 1978). Since then, species-specific variations in the short repeat have been described (Henderson 1995). In addition, telomeres are polarized. The G-rich strand is longer than the C-rich strand in most species, resulting in a 3′ overhang (Henderson and Blackburn 1989). The telomere G-rich strand can also form G4-quadruplexes, short, four-stranded structural motifs in which four guanines lie in a planar arrangement, each hydrogen bonded to two neighbors (Marsh *et al.* 1995). G4-quadruplex elements are found at telomeres but also scattered throughout the genome. They can form in a variety of ways within or between strands of DNA or RNA and require disruption by helicases for efficient telomere DNA synthesis (reviewed by Lipps and Rhodes 2009). Another conserved telomeric structure is the T-loop, in which the 3′ overhang is inserted by means of strand displacement into the upstream double-stranded telomeric region. The resulting lariat-like T-loops can be visualized by electron microscopy in DNA preparations from animals and plants (Griffith *et al.* 1999; Cesare *et al.* 2003). T-loops are thought to stabilize the ends of the chromosomes and to prevent inappropriate DNA-repair activity (Smogorzewska *et al.* 2000).

Telomeres also contain non-DNA components, including two relatively well-characterized multisubunit protein complexes, shelterin and the Cdc13/Stn1/Ten1 (CST) complex (reviewed by De Lange 2005; Giraud-Panis *et al.* 2010). The evolutionarily conserved shelterin complex facilitates T-loop formation and regulates telomerase action at the telomere. The complex is composed of six different proteins: TRF1, TRF2, TIN2, RAP1, TPP1, and POT1 (Liu *et al.* 2004; Ye *et al.* 2004, reviewed by De Lange 2005). Some of the components of shelterin have known homologs in plants, whereas others do not (Bunch *et al.* 2005; Shakirov *et al.* 2005). At least two families of plant-telomere double-strand DNA-binding proteins, RTBP1/TRFL and SMH, have been identified as having TRF-like single-myb DNA-binding domains (Yu *et al.* 2000; Marian *et al.* 2003). Some of these putative homologs of mammalian TRF proteins exhibit protein-protein interactions with other telomeric proteins (Kuchar and Fajkus 2004; Schrumpfova *et al.* 2008). The CST complex, found in animals, yeast, and plants, functions to maintain telomeric integrity, structure, and uniform length (Miyake *et al.* 2009; Surovtseva *et al.* 2009). One highly regarded model for telomere length regulation involves a negative-feedback or telomere protein-counting mechanism in which the length of the telomere and the abundance of the associated proteins produce a *cis*-acting negative feedback signal for telomerase-mediated extension (Marcand *et al.* 1997; Van Steensel and De Lange 1997). In addition, many components of the DNA damage machinery are essential for suppression of recombination and access of telomerase to the telomeres (reviewed by Lamarche *et al.* 2010). Other factors that influence the length and maintenance of telomeres include gene products associated with DNA replication, telomerase regulation, and telomere-repeat-containing RNA (reviewed by De Boeck *et al.* 2009; see also Riha *et al.* 2006; Feuerhahn *et al.* 2010).

Most of what is known about plant telomere length control comes from genetic analyses in Arabidopsis that used knockout, knockdown, or overexpression of telomere-associated genes (for review see Watson and Riha 2010a), but the biological significance of naturally occurring telomere length variation in plants remains a mystery. The age-related telomere shortening observed in animals may not be a general feature of plants, as aging in plants is not directly comparable to aging in animals (for review, see Watson and Riha 2010b). Furthermore, the correlation between telomere length and age in plants seems to differ in different species. For example, barley telomeres shorten during embryonic and inflorescence development (Kilian *et al.* 1995), whereas Arabidopsis and *Melandrium album* telomeres showed little or no change during growth and development (Riha *et al.* 1998). In addition, long-lived pine trees have longer telomeres than some of their shorter-lived counterparts (Flanary and Kletetschka 2005), whereas Gingko trees exhibited slightly longer telomeres with increasing age and display seasonal fluctuations in telomere lengths (Liu *et al.* 2007; Song *et al.* 2010). These and other cases highlight the need for more information about the biological significance of heritable variation in telomere length.

We therefore initiated a quantitative trait locus (QTL) mapping study to identify loci that contribute to telomere length variation in maize (*Zea mays* L.). In a previous QTL mapping study of telomere length control in maize, several QTL were found to account for a significant proportion of telomere-length variation (Burr *et al.* 1992). The mapping population was relatively small, however, and these QTL intervals have not been further characterized (Burr *et al.* 1992; Knapp *et al.* 1992). In humans, QTL linkage analysis of telomere length control led to the identification and analysis of several candidate genes, including the DDX11 helicase (Vasa-Nicotera *et al.* 2005), Rad51L, and FANCD2 (Andrew *et al.* 2006). A similar analysis in *Saccharomyces cerevisiae* identified two major loci with candidate genes necessary for telomere maintenance, as previously demonstrated by deletion analysis (Askree *et al.* 2004; Gatbonton *et al.* 2006).

A well-developed and widely used mapping resource in maize is the recombinant inbred lines (RILs) of the maize IBM (intermated B73 × Mo17) population. It is composed of 302 RILs with over 2000 mapped genetic marker loci (Lee *et al.* 2002; Sharopova *et al.* 2002). This population has been successfully used to map control loci for variation in cell-wall composition (Hazen *et al.* 2003), pest and fungal resistance (Nair *et al.* 2005; Balint-Kurti *et al.* 2007; Ordas *et al.* 2009), plant architecture (Lauter *et al.* 2008), tassel architecture (Pressoir *et al.* 2009), and seedling biomass (Zhang *et al.* 2010). Here we report our findings from QTL mapping of telomere length-control factors in the maize IBM population as well as results from quantitative real-time PCR expression assays performed on 16 candidate genes.

## Materials and Methods

### Plant materials

Maize seed of the 302 F2 RILs of the IBM population were obtained from the Maize Genetics Cooperation Stock Center (http://maizecoop.cropsci.uiuc.edu/, Urbana, IL). The 302 maize diversity lines (Flint-Garcia *et al.* 2005) and the subset of 25 lines used as parents for the nested association mapping (NAM) population (McMullen *et al.* 2009) were obtained from the North Central Regional Plant Introduction Station (orders 185771, 179535, USDA-ARS, Ames, IA). For DNA, 3- to 5-cm-long primary or secondary immature ear shoots, mature leaves, or emerged predehiscent tassels were harvested from plants grown in the greenhouse or field at the Mission Road Research Facility of the Department of Biological Science, Florida State University, Tallahassee, FL. Seedlings for RNA were grown under fluorescent light banks indoors at 23° with 16 h light, 8 h dark, in superfine germinating mix (Fafard), and above-ground tissues were harvested at 2:00 pm, 2 weeks after planting. All plant material was quickly flash-frozen after harvest in liquid N_2_ and stored at –80° until use.

### DNA extraction

Plant tissues were ground frozen into a fine powder in liquid N_2_ with a mortar and pestle and kept at –80° until addition of the first DNA-extraction buffer. Total DNA was extracted by one of the following three methods: a CTAB/phenol extraction method based on that of Saghai-Maroof *et al.* (1984), an aqueous DNA extraction method from Dellaporta (1994), and a Qiagen DNeasy Plant Maxi kit method (Qiagen, #68163). Integrity of total DNA was examined by inspection of ethidium bromide–stained agarose gels after electrophoresis.

### TRF analysis

Total DNA (4–15 μg) was digested with 2.5 U/μg each of *Alu*I, *Hae*III, and *Mbo*I in 1× Buffer 2 (New England Biolabs) at 37° for 18 h. For Southern blot analysis, digested DNA was separated on large 0.8% agarose gels by electrophoresis. DNA gel blots were performed essentially as by Southern (1975) onto Nytran-SPC (Whatman) nylon membranes and UV crosslinked (120 mj/sec, BioRad Stratalinker). DNA from a telomere PCR product generated by means of a template-free reaction (Ijdo *et al.* 1991) was used for the telomere probe. The telomere PCR product and lambda DNA probes were made separately by random-primed labeling with ^32^P-dCTP and combined for the hybridization. Southern hybridization was performed at 68° with previously described aqueous buffers (Bass *et al.* 1994), and the blots were exposed to a PhosphorScreen (GE Healthcare Life Sciences) and imaged on a Typhoon Imaging System (GE Healthcare Life Sciences).

### QTL mapping

PhosphorImages were analyzed with ImageQuantTL (GE Healthcare Life Sciences) with the λ-*Hin*dIII molecular-weight marker to yield a standard curve. For median and mean telomere-length measurements, the radioactive signal intensity for each lane was divided by the molecular weight, and the resulting size-adjusted frequency distribution was subjected to statistical analysis with JMP 8 Statistical Software (SAS Institute).

Composite interval mapping (CIM; Zeng 1993) of telomere length data was implemented in QTL Cartographer v1.17f (Basten *et al.* 2000). The genetic marker data set included 2019 markers (Sharopova *et al.* 2002; Lauter *et al.* 2008) for which quality-control tests of line identity had been performed (Lauter *et al.* 2008). On the basis of the quality-control data (not shown), 17 of the 195 phenotyped IBM RILs were omitted from our analysis. CIM estimations of QTL likelihoods and effects were performed at ∼8000 test positions at 1-centiMorgan (cM) intervals with five genetic markers as cofactors, which were selected by forward stepwise regression. A 10-cM blockout window on each side of the test position was imposed to allow local cofactor exclusion.

To limit false discovery of QTL, we performed permutation tests to establish an α = 0.01 comparisonwise threshold (CWT) for each test position (Doerge and Churchill 1996). For each of 1000 permutations of the phenotypic data, CIM was performed with the same parameters and steps, including cofactor selection as described by Lauter *et al.* (2008). For each trait at each test position, the 11^th^ highest likelihood-ratio test-statistic value (LRTS) observed was used to set the CWT for that position. Only QTL deemed to be statistically significant by this method are reported.

For each QTL, a support interval (SI) based on the “2-logarithm-of-odds drop” method is reported. These SIs approximate a 95% confidence interval for the positional localization of each QTL (Mangin *et al.* 1994; Crossett *et al.* 2010). The SIs were obtained from the map coordinates at which the LRTS had dropped 9.21 units from the QTL peak, which is equivalent to two logarithm-of-odds ratio units [logarithm of odds = LRTS/(2 × ln10) = 4.605]. SI boundaries were also inferred if the LRTS dropped below 0.25 or if the end of a chromosome was reached. SI boundaries would have been inferred where a change in additive-effect direction occurred (supporting information, Figure S1), but no such changes occurred within the initial SIs recorded.

### Estimation of QTL effects by marker regressions

To minimize CIM estimate–based inflation of QTL effects (Zeng 1993; Beavis *et al.* 1994), we performed regressions with the genetic markers nearest to the detected QTL. Regression models were built by iterative testing of the fit of each marker. For median telomere length QTL (TEL-MD), the QTL bins (and their respective markers) were 2.09 (*bnlg469b*), 3.06 (*AI714716*), 4.01 (*umc1682*), 4.03 (*npi386*), 5.03 (*umc1315*), 5.04 (*umc1966*), 6.04 (*umc65a*), 7.04 (*umc1708*), 9.02 (*umc1636*), and 10.01 (*AW330564*). For mean telomere length QTL (TEL-MN), they were 1.01 (*umc1906*), 1.05 (*umc1977*), 2.09 (*bnlg469b*), 3.06 (*AI714716*), 4.01 (*umc1682*), 4.03 (*npi386*), 5.03 (*umc2295*), 6.05 (*uaz121a*), and 7.04 (*AY110439*). Within a bin, the two traits may differ in the marker selected to represent a QTL because the closest marker to the QTL peak was used. For example, bin 5.03 is represented by *umc1315* for TEL-MD and by *umc2295* for TEL-MN. Terms were only retained in the final regression model for a trait if they were significant at the α = 0.05 level and their exclusion caused a drop in the adjusted R^2^ value for the model.

### Testing for QTL-by-QTL interactions

Using only the markers judged significant by regression criteria, we performed pairwise tests for interaction using two-way analysis of variance. For both traits, 21 tests were performed among the seven significant markers that acted as surrogates for the QTL. To limit false discovery associated with multiple testing, we used Q-value (Storey 2002; Storey and Tibshirani 2003) to evaluate the significance of *P*-values among the 21 tests for each trait (false-discovery rate of 10%). An interaction effect is reported only if it met this criterion and could be added to the regression model under the criteria described above.

### Bioinformatic screen for candidate genes

Candidate genes were identified by inspection of the genome annotations and gene models from the reference genome of B73 (AGPv2 at http://maizesequence.org and http://maizegdb.org; Schnable *et al.* 2009), which is one of the two IBM parental inbred lines. Genomic regions primarily within and occasionally adjacent to the QTL peaks with relatively narrow support intervals were examined. Depending on interval size and gene density, different QTL peaks were typically associated with 3–10 initial candidates, a list narrowed to a top-ranked choice for each locus by several criteria. The relative rankings of candidate genes were elevated if (1) they were already known to affect telomere metabolism or length in other species (*e.g.*, see Askree *et al.* 2004; Gatbonton *et al.* 2006), (2) they encoded proteins with biochemically defined telomere DNA binding activity (*e.g.*, see Dejardin and Kingston 2009), (3) they encoded proteins known to be involved in DNA repair or replication, or (4) they were associated with expression or regulation of telomerase.

### Real-time qPCR

mRNA was purified from 16 genotypes with telomere lengths spanning the range of each population. For the IBM mapping population, these were Mo197 (2.4 kb), Mo362 (3.6 kb), Mo373 (3.7 kb), Mo283 (4.2 kb), Mo210 (7.3 kb), Mo248 (16.2 kb), Mo335 (16.5 kb), and Mo321 (22 kb). For the maize diversity lines, these were Mo18w (2.5 kb), Ki11 (2.6 kb), B73 (3.2 kb), NC358 (4.1 kb), Mo17 (8.8 kb), Oh43 (12.3 kb), IL14H (14 kb), and M37w (16 kb). RNA was extracted with the RNeasy Plant Mini Kit (Qiagen) and reverse transcribed into cDNA with SuperScript III (Invitrogen). Gene-specific primers (Table S3) were designed for each candidate gene based on the cDNA or gene model sequences from http://maizesequence.org. Most of the RT-PCR products, 50–170 bp long, were designed to span an intron, providing an internal control for detection of contaminating genomic DNA. RT-PCR products were cloned and sequence-verified for each target gene. Quantitative real-time PCR was performed on three biological replicates with an ABI 7500 Fast machine and SYBR Green PCR Master Mix (Applied Biosystems). Melt curves were examined for problems associated with genomic DNA contamination, primer-dimers, or multiple products. Suitable targets were then used in a template dilution series to optimize for reaction efficiency. Cycle threshold values for each RIL were normalized to cytosolic *Gapdh* (GRMZM2G046804, http://maizesequence.org).

## Results and Discussion

### TRF analysis of maize telomere length

**Figure 1:**
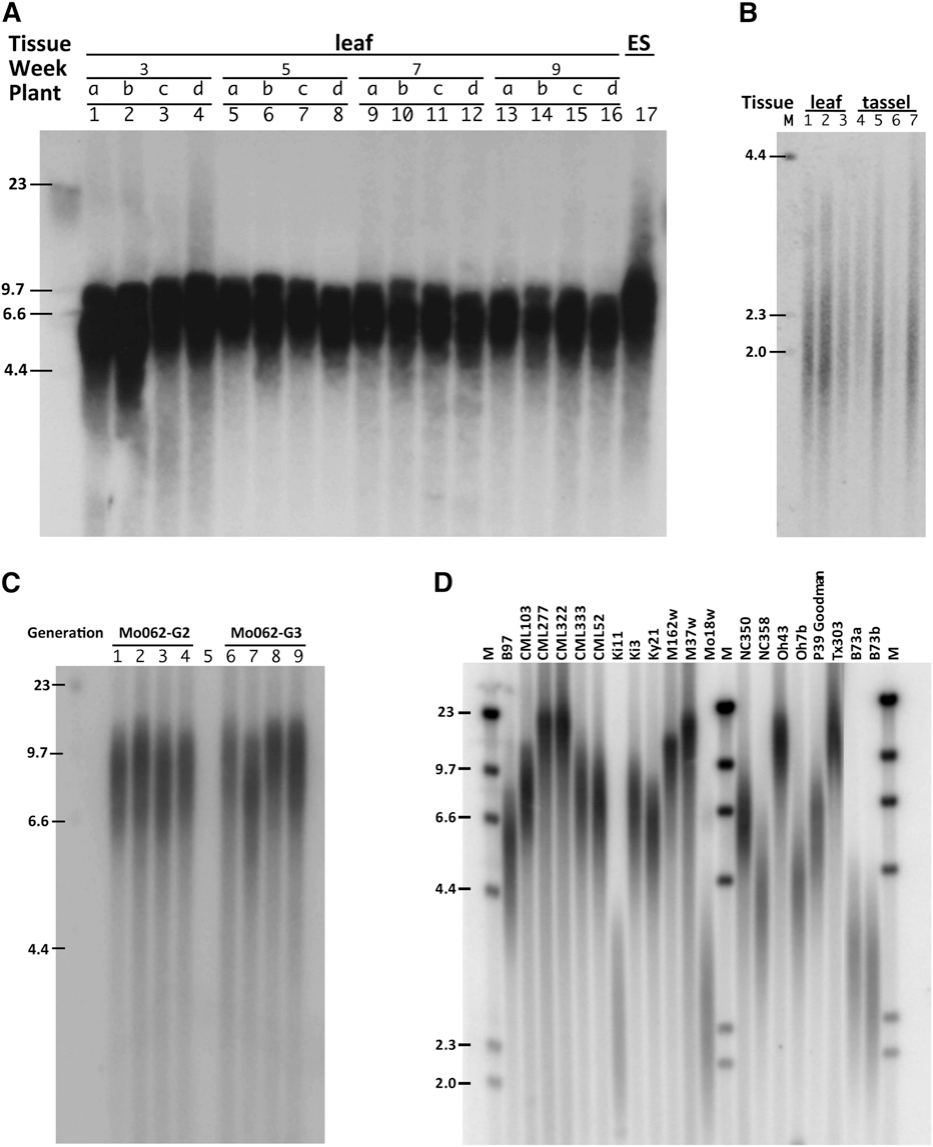
TRF analysis of maize telomere length. Southern blots of various tissues and developmental stages were hybridized with a telomere-repeat probe. (A) Leaf DNA from four Mo005 siblings (plants A–D) at weeks 3, 5, 7, and 9 after planting and 3–5 cm immature ear-shoot (ES) DNA (lane 17). (B) Leaf DNA (lanes 1–3) and emerged predehiscent tassel DNA (lanes 4–7) from B73 siblings. (C) Three- to five-centimeter ear-shoot DNA from four siblings (lanes 1–4 and 6–9) from each of two generations (G2 and G3) of RIL Mo062. (D) A collection of maize inbred lines selected for their genetic diversity. The genotype is noted above each lane. M indicates the lanes with the molecular size marker, λ-*Hin*dIII; sizes (kb) are indicated at the left side of each blot.

Figure 1 shows the results of our TRF analysis examining telomere length variation as a function of maize development and genotype. The mean telomere length from RIL-Mo005 siblings was maintained in the leaf samples throughout the growing season and differed little from that of the immature ear shoot, an organ with multiple reproductive meristems (Figure 1A, lane 17). A comparison of the telomere lengths of B73 for vegetative (leaf) and male reproductive (emerged, predehiscent tassels) tissues also revealed little variation (Figure 1B). In addition, telomere length appeared stable within individual RILs (individuals from RIL Mo062, lanes 1–4, 6–9, Figure 1C) and across generations (compare G2 and G3, Figure 1C).

The uniformity of telomere length in maize is remarkable, and the consistent length seen throughout the life cycle (Figure 1A) appears to contradict predictions based on the “end-replication problem” or the general animal model of age-associated telomere shortening. Telomeres may be shortening slightly with each cell division, but if so, the changes are too small to be detectable with this technique.

The short life cycle and unique properties of plant meristems in relation to organ age and development may also contribute to the line-specific uniformity in telomere length. Plant meristems are present at all growing tips of the plant throughout the life cycle and resemble animal embryonic cells in that they are proliferative tissues known to express relatively high levels of telomerase (Fitzgerald *et al.* 1996; Kilian *et al.* 1998). Different leaves initiated from the meristem may have undergone a similar number of cell divisions since organ initiation, despite their difference in apparent age. This aspect of plant development confounds the concept of age as having a linear relationship with time (Figure 1A; for review see Watson and Riha 2010b). Our findings are also consistent with the idea that a genotype-specific telomere-length “set point” is established to provide uniformity from one generation to the next (Figure 1C; Schaetzlein *et al.* 2004; Shakirov and Shippen 2004). All of these factors, along with the comparatively short life cycle of annual plants such as maize, may contribute to the uniformity of telomere length we observed.

In contrast to the high uniformity of telomere length within genotypes, variation among genotypes of maize is high (Burr *et al.* 1992). Large-scale analyses of diverse inbred maize lines has revealed a high degree of genotypic and phenotypic variation, allowing for the identification of a select subset of inbred lines of maize that capture much of the genetic variation found in the species (reviewed by Yu *et al.* 2008). For our study, we selected genotypes from among the 25 diverse lines that make up the parents of the NAM population (McMullen *et al.* 2009). From the TRF analysis of these diverse lines, we found more than 10-fold variation in telomere lengths, ranging from short (B73, 2.3 kb) to long (CML277, 19 kb), as shown in Figure 1D. We note that telomere length for a given line was also uniform when sampling tissues from different years, organs, or stock accessions, as summarized in Table 1. The variation we see in maize is remarkable in that it resembles the amount of variation normally associated with the entire plant kingdom (Kilian *et al.* 1995; Fajkus *et al.* 1995, 2002; Shakirov and Shippen 2004). This pattern of line-specific uniformity and population-wide natural variation makes maize telomere length an ideal trait for genetic analysis.

**Table 1:** Telomere lengths of diverse maize lines

Line	Accession*^a^*	Telomere Length (kb)*^b^*	Tissue Source
B73a		2.8*^c^*	Leaf pool
B73b		3.2*^c^*	Leaf pool
B73	MDS	3.2	Immature ear shoot
B73	DL25	2.7	Immature ear shoot
B73	IBM	3.2	Leaf, tassel
CML103	MDS	9.2	Immature ear shoot
	DL25	7.3	Immature ear shoot
CML333	MDS	6.8	Immature ear shoot
	DL25	6.2	Immature earshoot
HP301	MDS	2.2	Immature ear shoot
	DL25	2.8	Leaf, tassel
IDS28	MDS	7.2	Immature ear shoot
	DL25	6.2	Immature ear shoot
IL101	MDS	4.9	Immature ear shoot
	DL25	4.9	Immature ear shoot
Ky21	MDS	5.4	Immature ear shoot
	DL25	6.1	Immature ear shoot
Mo17		8.8*^c^*	Leaf pool
	IBM	9.4	Ear shoot
	DL25	9.4	Immature ear shoot
Mo18w	MDS	2.1	Immature ear shoot
	DL25	2.5	Immature ear shoot
NC348	DL25	4.4	Immature ear shoot
	MDS	4.5	Immature ear shoot
Oh43		10.6*^c^*	Leaf pool
	DL25	12.3	Immature ear shoot
Oh43e	MDS	12.1	Immature ear shoot
	DL25	14.3	Immature ear shoot
Pa91	MDS	4.9	Immature ear shoot
	DL25	4.7	Immature ear shoot
T232		39.4*^c^*	Leaf pool
	DL25	>24	Immature ear shoot
Tx601	DL25	11	Immature ear shoot
	MDS	10.5	Immature ear shoot

a In-house accession of source seed for the genotype listed. MDS = maize diversity set (of 302); DL25 = 25 diverse lines/NAM parents (McMullen *et al.* 2009).

b Determined as described in *Materials and Methods*, with the exception of the Burr *et al.* (1992) data.

c From data of Burr *et al.* (1992).

### TRF analysis of the maize IBM population

**Figure 2:**
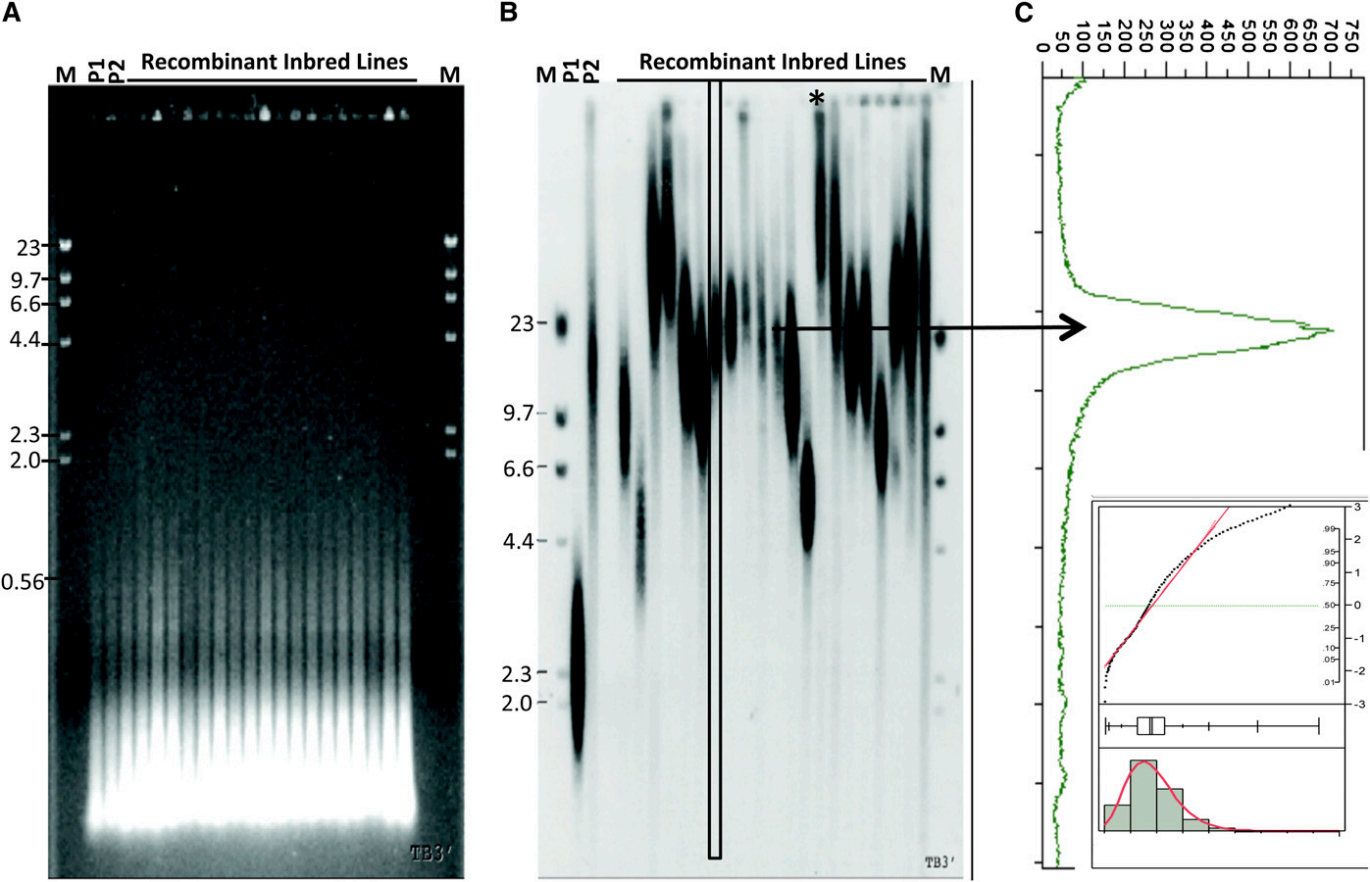
TRF analysis of the maize IBM population. Shown are representative ethidium-bromide–stained agarose gel (A) and TRF blot (B) of the two parents and 24 recombinant inbred lines from the IBM population. TRFs were separated on an agarose gel, transferred to a membrane, incubated with a ^32^P-labeled telomere-repeat probe, and visualized by phosphorimaging. (C) Densitometry data were used to calculate median and mean telomere lengths (arrow). P1 is the maternal parent, B73. P2 is the paternal parent, Mo17. M indicates the molecular size marker, λ-*Hin*dIII. Sizes (kb) are indicated at the left side of each blot. The asterisk indicates data omitted from the analysis.

Our QTL mapping study with the IBM population (Lee *et al.* 2002), integrated with the annotated B73 genome, permits fine mapping and rapid development of candidate genes for downstream analysis. The general telomere-length phenotyping strategy we employed is summarized in Figure 2. We chose immature ear shoot for the DNA source because it yields large amounts of high-quality DNA and exhibits telomere lengths similar to those of other tissues in a given genotype (see Figure 1A–C; Table 1). Total maize DNA digested with a cocktail of restriction enzymes produces a pattern of restriction fragments mostly less than 500 bp long (Figure 2A). Telomere lengths vary greatly among RILs within the IBM population (Figure 2B), exhibiting transgressive segregation. For any particular RIL, the TRF signal on the blots (Figure 2B) represents a population of fragments that yield broad or smeared-looking bands in each lane. This band morphology is typical for TRF assays and results primarily from the inherent variation in telomere tract length found at the 40 different telomeres in diploid maize (2n = 2x = 20). Other contributing factors may include variation in subtelomeric DNA sequence where the restriction enzyme cuts and cell-type variation within the tissue. Despite this variation, each line produces a population of telomeres that is apparently maintained around a genotype-specific set point, which can be quantified and represented by a single value, such as the median or mean length.

The procedure for measuring the average telomere length employed single-lane signal-intensity scans (Figure 2B, arrow) that were assigned a base-pair size by means of the λ-*Hin*dIII standard curve and converted to signal-distribution histograms (Figure 2C, arrow). These were subjected to statistical analysis that yielded the median and mean telomere length. The resulting size-normalized median and mean telomere lengths (for 178 IBM RIL lines, listed in Table S1)reveal size variation ranging from 3.2 kb to more than 20 kb, a difference of nearly an order of magnitude. In addition, some signals (Figure 2B, asterisk) were detected near the top of the gel, at a position representing a size larger than that of the DNA fragments isolated. The positions of these signals were attributed to aberrant migration due to the formation of G4-quadruplex DNA structures formation. G4-quadruplex DNA is known to form in response to heating, the presence of potassium ions, high concentrations of DNA, and even repeated freezing and thawing of samples (Henderson *et al.* 1987; Marsh and Henderson 1994; Marsh *et al.* 1995; Penazova and Vorlickova 1997). We found that individual DNA preparations showing aberrant migration patterns were resistant to denaturation (data not shown), and such preparations were not used in the determination of line-specific telomere lengths.

### Detection and characterization of QTL effects

**Figure 3:**
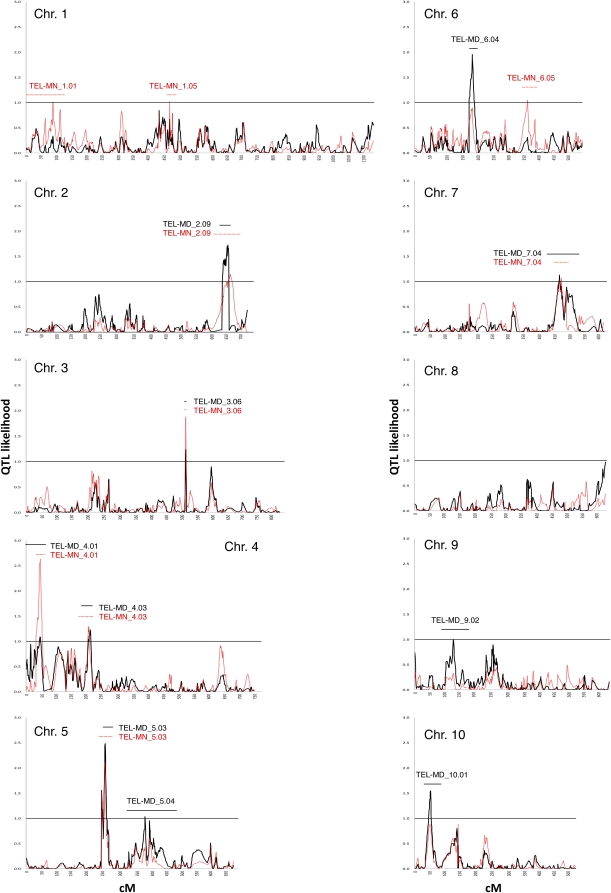
Quantitative trait locus (QTL) mapping of median and mean telomere length. The X-axis represents centiMorgans (cM) along each chromosome, and the Y-axis represents the QTL likelihood. The bar at likelihood = 1 indicates the normalized threshold for significance of the median (black line) and mean (red line) telomere-length QTL. The chromosome number (Chr) and QTL names (TEL-MD and TEL-MN) are indicated for each plot.

Size-normalized median and mean telomere lengths were both subjected to CIM with a high-resolution linkage map that is 7090 cM long and contains 2019 markers (Sharopova *et al.* 2002). QTL were robustly detected for both traits as shown in the QTL likelihood plots in Figure 3. The QTL that exceeded an α = 0.01 comparisonwise significance threshold are reported in Table 2 and named according to the genetic linkage bin in which they reside (Davis *et al.* 1999). Ten median and nine mean QTL were detected; six loci (2.09, 3.06, 4.01, 4.03, 5.03, and 7.04) exceeded the significance threshold for both traits (Table 2). For the seven cases where the LRTS for only one trait exceeded the significance threshold, the LRTS for the other trait often exhibited a similar shape, suggestive of a weak effect rather than a functional difference. This pattern is most clearly seen for the QTL on chromosomes 6, 9, and 10 (Figure 3).

**Table 2:** Telomere-length quantitative trait loci (QTL) from the maize intermated B73 × Mo17 (IBM) population

QTL Name*^a^*	IBM Map Position (cM)	LRTS*^b^*	CWT*^c^*	Additive Effect*^d^*	R^2^*^e^*	Support Interval*^f^*
TEL-MN_1.01	89	5.76	5.71	−386.00	0.024	0–127
TEL-MN_1.05	467	5.52	5.37	420	0.026	459–481
TEL-MD_2.09	652	13.87	9.44	−619	0.068	637–661
TEL-MN_2.09	652	9.13	9.05	−517	0.046	604–701
TEL-MD_3.06	513	10.57	8.54	−504	0.047	512–517
TEL-MN_3.06	513	9.79	10.11	−524	0.066	512–516
TEL-MD_4.01	43	7.54	7.34	457	0.037	0–57
TEL-MN_4.01	45	13.97	5.30	661	0.075	30–55
TEL-MD_4.03*^g^*	216	7.97	7.01	447	0.037	190–226
TEL-MN_4.03*^g^*	211	6.93	5.86	428	0.031	137–226
TEL-MD_5.03	260	15.13	6.09	−604	0.066	254–267
TEL-MN_5.03	259	13.69	7.05	−611	0.064	248–267
TEL-MD_5.04	377	6.74	6.52	397	0.028	319–470
TEL-MD_6.04	182	13.61	6.95	−579	0.059	171–193
TEL-MN_6.05	362	6.33	6.01	403	0.028	329–434
TEL-MD_7.04*^g^*	469	6.91	6.12	−450	0.036	428–533
TEL-MN_7.04*^g^*	473	8.34	7.84	−468	0.037	444–495
TEL-MD_9.02	131	6.90	6.91	406	0.029	93–190
TEL-MD_10.01	53	9.47	6.13	489	0.043	34–79

a Each QTL name comprises the trait identifier and the map region by chromosome (integer) and bin (decimal) numbers.

b LRTS = likelihood-ratio test statistic.

c CWT = Comparisonwise threshold exceeded by the QTL (α = 0.01; LRTS units).

d Estimate of the effect of one B73 allele at this locus, such that negative values indicate that Mo17 alleles add to telomere length.

e Proportion of the phenotypic variance explained by the QTL as estimated by composite interval mapping.

f Support intervals show the centiMorgan values at which the LRTS has dropped 9.21 units from its peak or below 0.25 for QTL peaks with LRTS values lower than 9.21, a decrease equivalent to two logarithm-of-odds units.

g A QTL position also reported by Knapp *et al.* (1992).

Marker regressions and conservative estimates of the median trait variance revealed that the QTL in bins 2.09, 3.06, 4.01, 5.04, 6.04, 9.02, and 10.01 accounted for 35.2% of the phenotypic variance. Similarly, analysis of mean trait variance revealed that the QTL in bins 1.01, 3.06, 4.01, 4.03, 5.03, 6.05, and 7.04 accounted for 33.1% of the phenotypic variance in telomere length. The partial R^2^ estimates for the individual QTL reflected the CIM results; no QTL effects explained more than 8% of the phenotypic variance. These regression analyses validated the CIM findings and provided the conservative estimates of aggregate QTL effects that explained about one third of the phenotypic variation for telomere length measurements.

Two-way analysis of variance among seven significant (see *Materials and Methods*) QTL for each trait revealed a significant interaction among the median QTL (5.04 and 2.09). The statistical significance of the interaction was confirmed by addition of the appropriate interaction term to the TEL-MD regression model. When it was added, the model explained 38.0% of the variance for TEL-MD, and the interaction term accounted for as much variance as either of the two weakest effects, about 3%. One interpretation of this interaction is that B73 alleles at the 5.04 QTL act epistatically to reduce the effects of the 2.09 QTL (Table 2). The least-squares means for TEL-MD among lines with Mo17 alleles at 2.09 and 5.04 is ∼6600 bp. Adding B73 alleles only at 5.04 does not alter TEL-MD significantly. When B73 alleles are present at 2.09 but not at 5.04, TEL-MD is reduced to 4600 bp, but when they are present at both loci, TEL-MD is not significantly different from 6600 bp. Another interpretation of this interaction is that Mo17 alleles at 5.04 are required to potentiate reduction of telomere length by B73 alleles, which would be consistent with CIM results that revealed a negative effect of Mo17 alleles at 5.04 (Table 2).

We note that B73 telomeres are among the shortest in maize. We therefore expected that, in the IBM population, alleles contributing to longer telomeres would be predominantly from Mo17. Surprisingly, this pattern was not generally observed. In fact, more than half of the QTL showed that the B73 allele promoted greater telomere length, but these loci have smaller effects on telomere length than the loci where Mo17 alleles were associated with longer telomeres (*e.g.* 2.09, 3.06, and 5.03). In addition, interaction effects such as the one described above can alter the relative strengths of genetic effects according to context. In this case, the strong negative impact of B73 alleles at 2.09 could be masked by the presence of the epistatic B73 alleles at 5.04.

In comparing our results with those of the previous maize telomere length QTL-mapping study, we found two loci common to the two: 4.03 and 7.04 [*ZP1F-UMC31A* and *BNL8.21-BNL8.39*, respectively, from Knapp *et al.* (1992); based on telomere-length data from Burr *et al.* (1992))], but our analysis did not detect any of the three previously described loci with the strongest effects [linked to markers *GLN1* on 10S, *BNL8.23* on 4L, and *YNH20* on 1L; (Burr *et al.* 1992)]. The most likely explanation for these differences is that we used different mapping populations.

### Expression analysis of candidate genes for telomere length control

The eight candidate genes used for expression analysis are listed in Table 3. Because of the multiple QTL and relatively long list of candidates, we used expression analysis to screen them, on the assumption that some phenotypic variation might result from expression-level differences governed by *cis*-acting elements affecting the candidate gene. Because any such expression variation may be small, we used the sensitive and quantitative qRT-PCR assay. This approach has been useful in identifying genes that contribute to variation in quantitative traits (Brown *et al.* 2005; Norry *et al.* 2009). We also examined expression variation of eight additional genes known *a priori* to be telomere-regulating genes.

**Table 3:** Target genes for qPCR analysis

Gene Name	Candidate Type	Telomere Relevance	Reference
*Putative Est1*	*a priori*	Est1-deficient *Saccharomyces cerevisiae* displays progressively shortening telomeres and increased chromosome loss.	Lundblad and Szostak (1989)
*Ibp2*	*a priori*	RTBP1, founding member of IBP/RTBP1/TRFL family, binds double-stranded telomeric DNA *in vitro* and decreases telomere length.	Yu *et al.* (2000), Yang *et al.* (2004)
*Ku70*	*a priori*	Ku70-deficient Arabidopsis displays telomere deregulation and lengthening.	Riha *et al.* (2002)
*Ku80*	*a priori*	Ku80-deficient Arabidopsis displays telomere deregulation and lengthening in a telomerase-dependent manner.	Gallego *et al.* (2003)
*Smh3*	*a priori*	A maize SMH family member binds double-stranded telomere repeats *in vitro*; Arabidopsis homolog interacts with POT1.	Marian *et al.* (2003), Kuchar and Fajkus (2004)
*Smh4*	*a priori*	A maize SMH family member binds double-stranded telomere repeats *in vitro*; Arabidopsis homolog interacts with POT1.	Marian *et al.* (2003), Kuchar and Fajkus (2004)
*Smh6*	*a priori*	A maize SMH family member binds double-stranded telomere repeats *in vitro*; Arabidopsis homolog interacts with POT1.	Marian *et al.* (2003), Kuchar and Fajkus (2004)
*Tert*	*a priori*	Telomere terminal transferase adds telomere repeats to telomeric DNA.	Greider and Blackburn (1985)
*Putative Rfc*	TEL_2.09	Mutation in large subunit of Replication Factor C causes a significant increase in telomere length in *S. cerevisiae*.	Adams and Holm (1996)
*Putative Mcm*	TEL_3.06	Human TRF2 stimulates ORC and MCM binding to telomeric chromatin.	Tatsumi *et al.* (2008)
*Hsp70-like*	TEL_4.01	Knock-out Hsp70^−/−^ MEF decreases telomerase expression, causes telomere loss, and increases end-to-end fusions.	Hunt *et al.* (2004)
*RecQL*	TEL_4.01	Dominant-negative WRN, a RecQ-like helicase, causes telomere loss and genome instability.	Bai and Murnane (2003)
*Putative Rpa 32*	TEL_5.03	Mutation in large subunit of Replication Protein A causes a significant decrease in telomere length in *S. cerevisiae*.	Ono *et al.* (2003)
*Smc5-like*	TEL_5.03	Inhibition of SMC5/6 in ALT cells inhibits HR and causes telomere shortening.	Potts and Yu (2007)
*Parp-like*	TEL_5.03	Tankyrase, a PARP, ADP-ribosylates TRF1, decreasing its affinity for telomeric DNA.	Smith *et al.* (1998)
*Rad51-like*	TEL_5.04	RAD51-deficient MEF exhibit shortened telomeres and increases in chromosome fusions.	Tarsounas *et al.* (2004)

**Figure 4:**
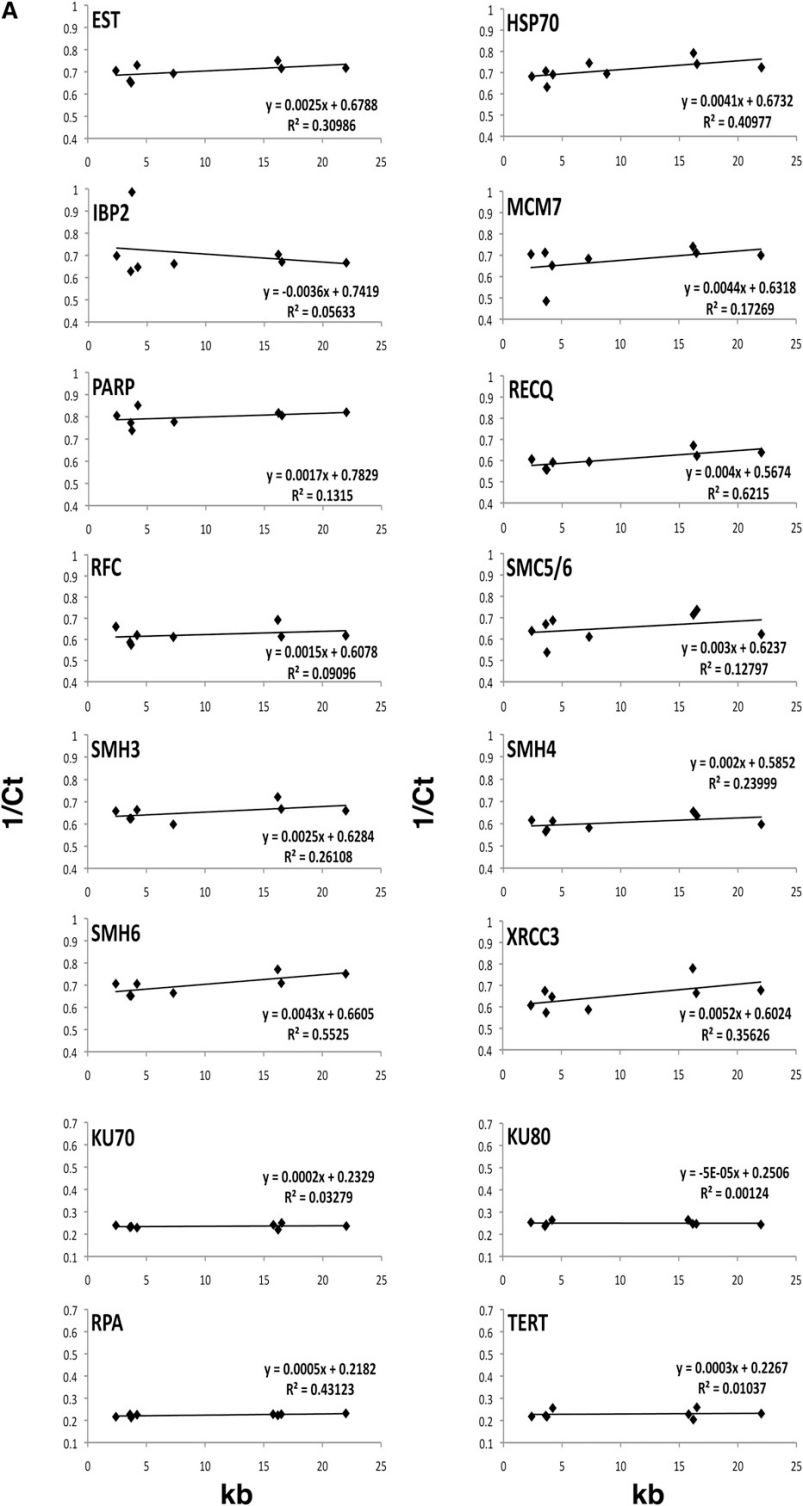

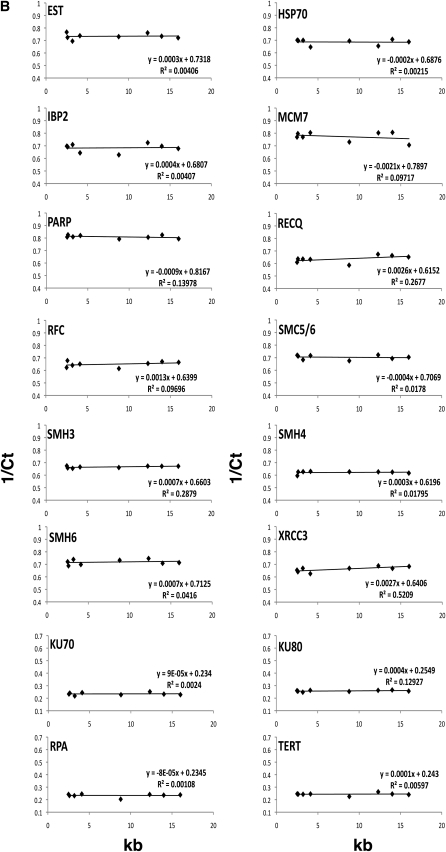
qPCR analysis of candidate gene expression in eight long- or short-telomere lines from the IBM population (A) and the diverse maize lines (B). *Gapdh*-normalized inverse cycle-threshold (Ct) values (Y-axis) are plotted against telomere length in kilobases (X-axis). Slopes and R^2^ values are indicated. The full gene name is given in Table 3. The gene model IDs are given in Table S3.

mRNA was isolated from maize seedlings, and the relative transcript abundance for target genes was determined by qRT-PCR. We examined eight RILs and eight diverse maize lines whose telomere lengths showed high variation. The gene-expression levels and telomere lengths, analyzed by means of bivariate correlation analysis, are plotted in Figure 4 for each gene. The normalized cycle-threshold values and standard deviations of the three biological replicates are reported in Table S2. For the IBM RILs (Figure 4A), the expression levels showed slight covariation with telomere length for some, but not all, candidate genes. The most common correlation was positive, and those that showed R^2^ values over 0.3 are *putative Est1*, *Hsp70-like*, *RecQL*, *Smh6*, and *Rad51-like*. Only one gene, *Ibp2*, showed a negative correlation. For the diverse lines (Figure 4B), even fewer correlations were observed. The two genes showing positive correlations in the diversity experiments were *RecQL* and *Rad51-like*, whereas none of the genes showed a significant negative correlation.

Although the slopes of the regression lines were small, all but one of the genes showing positive correlations have been previously established to be positive regulators of telomere length; the exception was *Smh6*, whose role in telomere length is unknown (Table 3, see references). Our results therefore suggest a possible role for the plant SMH (TRF-related) proteins as positive regulators of telomere length. Relatedly, mammalian TRF1 recruits POT1 to the telomeres, where it caps the 3′ end (Loayza and De Lange 2003). In plants, Arabidopsis SMH telomere-binding proteins have been shown by means of yeast two-hybrid assays to associate with plant homologs of POT1 (Kuchar and Fajkus 2004; Schrumpfova *et al.* 2008). Furthermore, POT1 proteins from several plants bind single-stranded telomeric DNA *in vitro* (Shakirov *et al.* 2009, 2010), whereas other members of the plant POT1 family, notably *At*POT1, appear instead to interact with the telomerase RNA (Cifuentes-Rojas *et al.* 2011). These observations, taken together with our findings, support a role for SMH proteins as positive regulators of telomere length, possibly affecting telomeres through direct DNA binding, by interactions with plant POT proteins, or both.

The negative correlation in the IBM lines of *Ibp2* is consistent with the earlier demonstration that rice and tobacco members of this RTBP/TRFL gene family are negative regulators of telomere length (Yang *et al.* 2004; Moriguchi *et al.* 2006; Hong *et al.* 2007). To date, only the RTBP/TRFL and SMH families of proteins in plants are known to encode double-stranded telomere DNA-binding proteins. A recombinant C-terminal myb-containing portion of *Zm*IBP2 also binds to telomere repeat DNA *in vitro* (J. M. Moore and H. W. Bass, unpublished observations). These results suggest one possible model for telomere length regulation in plants in which SMH and RTBP/TRFL have opposing roles in controlling overall telomere length.

Of all the candidate genes examined, the *Rad51-like* gene (TEL-MD_5.04) stands out in several respects. First, its QTL showed an epistatic interaction with another locus (TEL-MD_2.09), which maps to the same interval as a candidate gene with similarity to replication factor C (putative RFC). Both candidates belong to gene families associated with DNA metabolism, and a genetic interaction seems plausible. Second, the *Rad51-like* candidate gene showed the strongest correlation in the expression analysis in both the IBM RILs (Figure 4A) and the diverse lines (Figure 4B). Third, *RAD51L* was a candidate gene suggested by a human telomere-length QTL study (Andrew *et al.* 2006). Fourth, EST sequence analysis shows this gene (NCBI UniGene Zm.24480) to be expressed at 6, 21, and 201 transcripts/million in shoot, meristem, and embryo, all tissues where telomere set points or length maintenance is expected to be important. Finally, a role for RAD51-like proteins has been established for homologous recombination-based telomere maintenance (Tarsounas *et al.* 2004; Oganesian and Karlseder 2011). This class of proteins could potentially influence telomere length through its roles in multiple recombination-based pathways, which are known to play roles in telomerase regulation (Lamarche *et al.* 2010). Taken together, this information points to the *Rad51-like* gene TEL-MD_5.04 as a promising candidate for additional examination.

Interestingly, the diverse lines of maize show even less expression variation than the IBM RILs in our qPCR profiling experiments, even though they represent a sampling of up to eight alleles per locus. Several explanations are possible; for example, the diversity lines examined may have spanned less phenotypic space (2.5–16 kb) than the IBM RILs (2.4–22 kb). In this case, the inclusion of lines with larger TRFs may increase our ability to detect correlations. In addition, a lack of correlation in mRNA abundance and telomere length in both the IBM and diversity-line experiments could have several explanations: variation in protein sequence or activity that is not associated with transcript abundance, linkage of a candidate that is not the causal gene for the QTL, or relatively low variation of maize telomere-regulating genes. Indeed, genome-wide association studies in humans found that 37 telomere-maintenance genes analyzed show limited genetic variation, high ancestral allele frequencies, and low population differentiation (Mirabello *et al.* 2010). These results suggest that human telomere-maintenance genes are under selective pressure against extensive evolutionary divergence.

The remarkably high natural genetic variation in maize should be ideally suited for mapping low-variance telomere-maintenance genes in mapping populations such as the recently developed NAM population (Gore *et al.* 2009; McMullen *et al.* 2009). The study reported here revealed several regions of the maize genome that harbor allelic variation associated with telomere-length regulation. Subsequent screening by expression analysis helped identify specific genes for further study. Overall, we find that quantitative genetic analysis of naturally occurring telomere-length variation in maize has excellent potential to shed light on telomere-length control in plants, an important phenomenon from both evolutionary and agronomic perspectives.

## Supplementary Material

Supporting Information
