# Supplementary material for: QTL Mapping and Candidate Gene Analysis of Telomere Length Control Factors in Maize (Zea mays L.)
Source: G3 (Bethesda). 2011 Nov 1;1(6):437–50. doi: 10.1534/g3.111.000703 (PMC3276162; doi:10.1534/g3.111.000703)
Supplement: Supporting Information [file supp_1_6_437__index.html]

Supporting Information 

# QTL Mapping and Candidate Gene Analysis of Telomere Length Control Factors in Maize (*Zea mays* L.)

## Supporting Infomation for Brown *et al.*, 2011

**Files in this Data Supplement:**

- Supporting Information - Figure S1 and Tables S1-S3 (PDF, 1.6 MB)
- Figure S1 - A) Black lines represent the QTL Likelihood for TEL\_MD and the comparison-wise significance threshold (α = 0.01) at y=1 (left axis) B)Red lines represent the QTL Likelihood for TEL\_MN and the comparison-wise significance threshold (α = 0.01) at y=1 (left axis) (PDF, 1.3 MB)
- Table S1 - Telomere lengths for maize intermated B73 � Mo17 (IBM) recombinant inbred lines used for quantitative-trait-locus analysis (PDF, 52 KB)
- Table S2 - Normalized cycle-threshold values and standard deviations (three biological replicates) for eight IBM RILs and eight diverse maize lines (PDF, 128 KB)
- Table S3 - Primers for qPCR analysis (PDF, 72 KB)
