# Supplementary material for: QTL Mapping and Candidate Gene Analysis of Telomere Length Control Factors in Maize (Zea mays L.)
Source: G3 (Bethesda). 2011 Nov 1;1(6):437–50. doi: 10.1534/g3.111.000703 (PMC3276162; doi:10.1534/g3.111.000703)
Supplement: Supporting Information [file supp_1.6.437_000703SI.pdf]

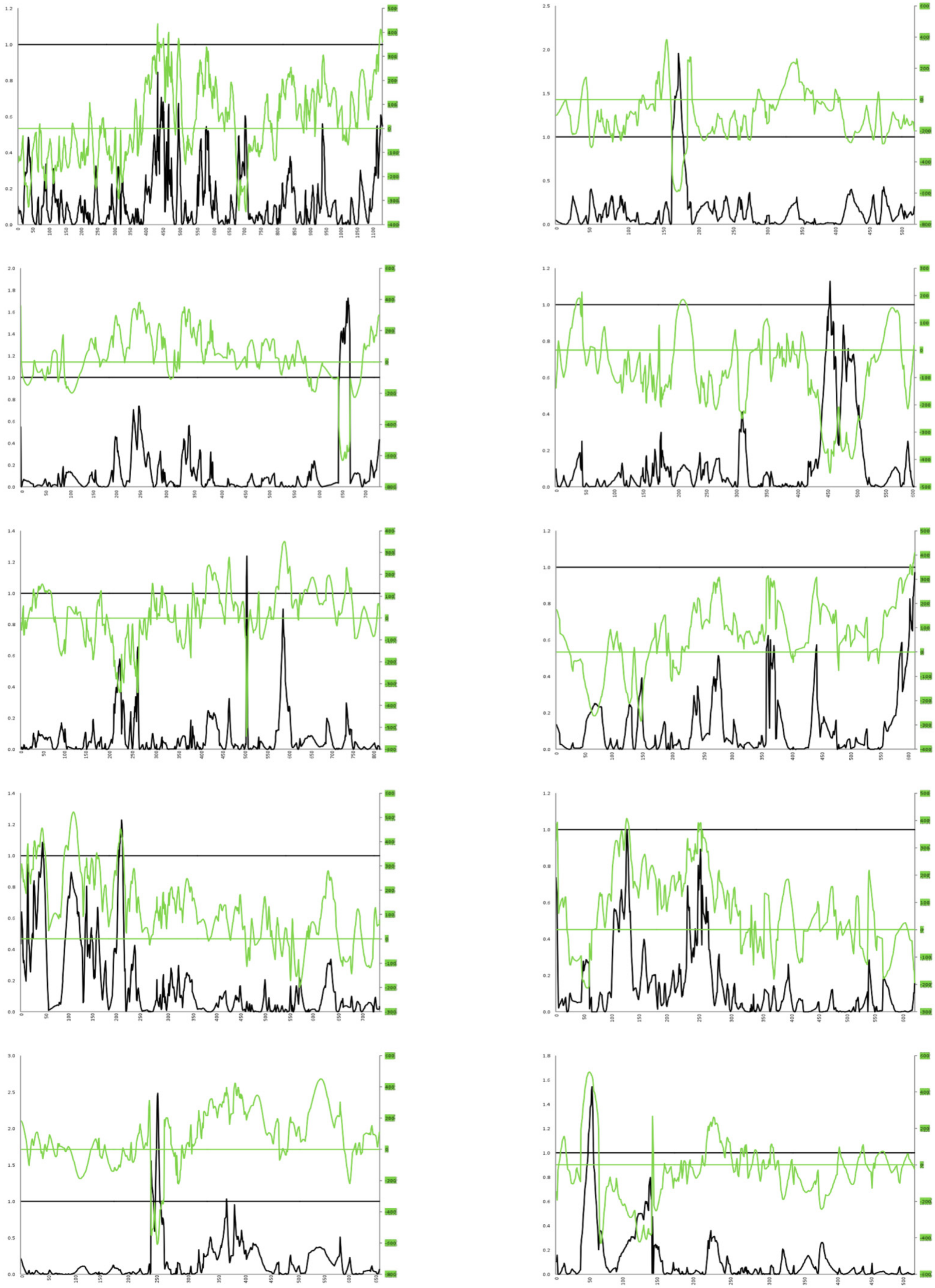

Figure S1A Black lines represent the QTL Likelihood for TEL_MD and the comparison-wise significance threshold (α = 0.01) at y=1 (left axis). Green lines represent the additive effect estimate and the boundary for changes in direction of effects at y=o (right green axis).

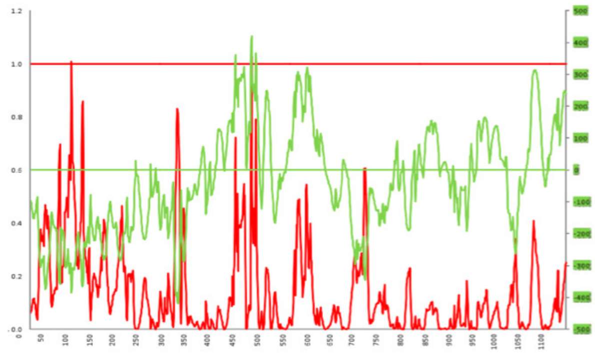
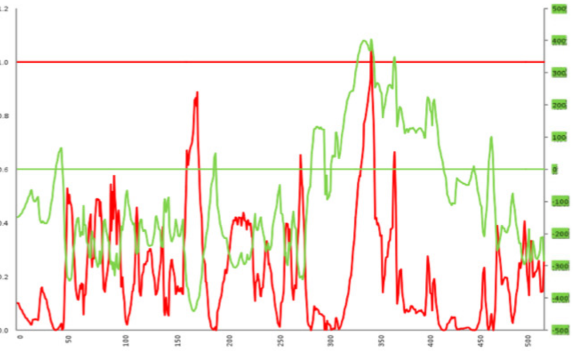
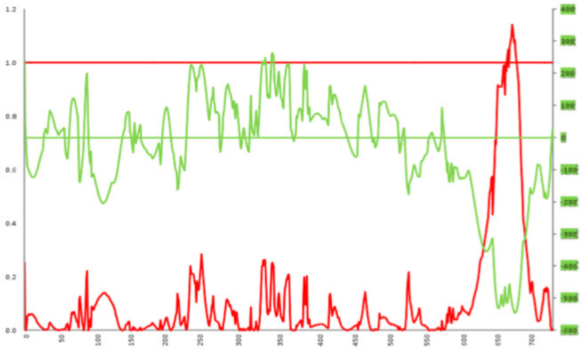
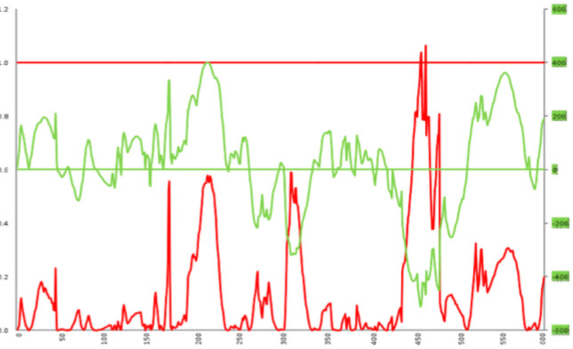
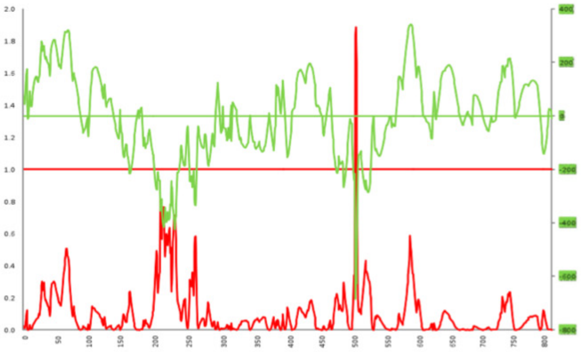
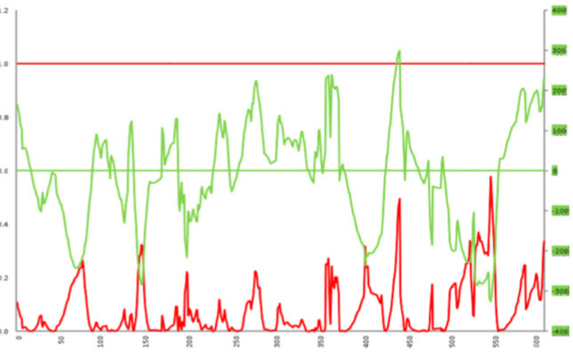
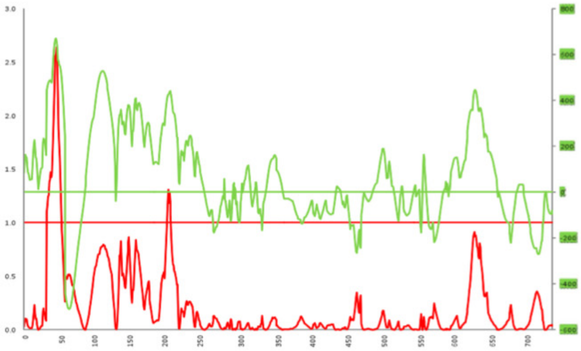
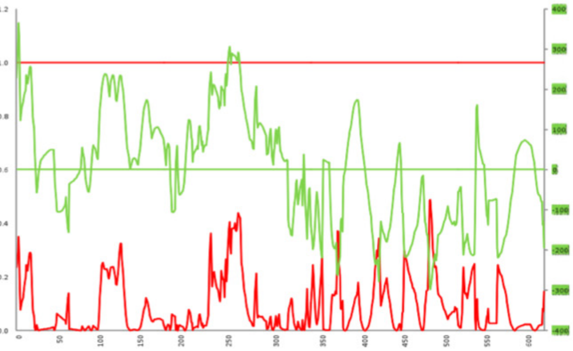
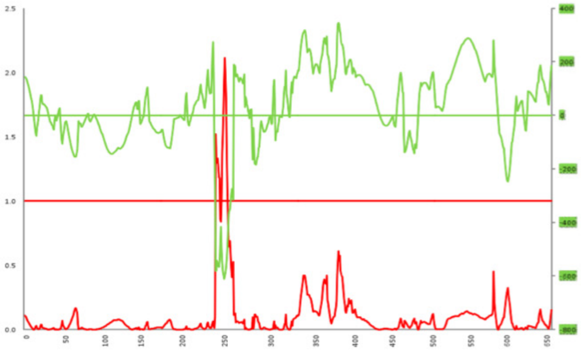
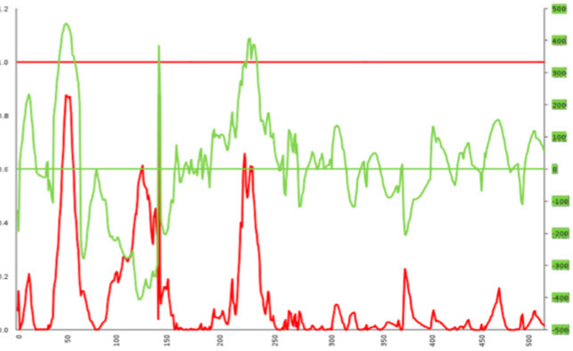

Figure S1B Red lines represent the QTL Likelihood for TEL_MN and the comparison-wise significance threshold (α = 0.01) at y=1 (left axis). Green lines represent the additive effect estimate and the boundary for changes in direction of effects at y=o (right green axis).

Table S1 Telomere lengths for maize intermated B73 × Mo17 (IBM) recombinant inbred lines used for quantitative-trait-locus analysis.

Line	TEL-MD	TEL-MN
Mo001	7266	7502
Mo003	5566	6385
Mo005	5874	5927
Mo007	7627	7314
Mo011	6490	6577
Mo012	5595	6089
Mo013	6011	6215
Mo014	6839	6424
Mo015	6545	6468
Mo016	7180	7984
Mo017	5829	6521
Mo018	6707	7001
Mo019	3649	4225
Mo021	4928	5173
Mo022	4505	4556
Mo023	7473	7765
Mo024	4164	4189
Mo025	6705	6842
Mo026	6246	6354
Mo027	6566	6693
Mo028	8413	9126
Mo029	3951	4364
Mo030	6395	6690
Mo034	6171	8928
Mo038	3976	4572
Mo039	4575	4905
Mo040	11400	11802
Mo041	5610	5777
Mo042	5836	5896
Mo043	6501	7580
Mo044	8209	8906
Mo046	4897	4902
Mo047	4814	4860
Mo051	3937	4477
Mo052	5201	5330
Mo054	5117	5187

Mo055	3530	3668
Mo057	3742	3954
Mo058	6876	7269
Mo060	7741	8054
Mo061	3685	3816
Mo063	3555	3772
Mo067	5375	5555
Mo068	6274	7177
Mo071	7897	8738
Mo074	2770	2967
Mo080	5948	5977
Mo081	5503	5939
Mo083	5503	6561
Mo085	4264	4551
Mo092	2958	4136
Mo093	4905	5396
Mo096	7822	7860
Mo097	4915	4928
Mo106	5547	5531
Mo109	3332	3251
Mo111	1667	2437
Mo113	5443	5502
Mo114	5713	5759
Mo116	5713	5919
Mo118	5237	5653
Mo119	3555	3772
Mo120	7563	7916
Mo124	3951	4023
Mo127	3420	3607
Mo128	7047	7264
Mo130	4587	4930
Mo132	4756	4985
Mo134	3057	3140
Mo138	7123	7709
Mo141	4930	5626
Mo142	4755	5678
Mo143	5693	6429
Mo145	5298	5472
Mo146	4587	4676

Mo147	2968	3076
Mo150	8633	8477
Mo151	1956	3021
Mo153	4491	4467
Mo154	6168	6182
Mo156	6659	6975
Mo157	5713	5796
Mo159	3749	4037
Mo160	3343	4348
Mo164	4048	4213
Mo167	9513	9619
Mo168	6452	6871
Mo172	7536	7373
Mo174	7834	7980
Mo176	7249	7232
Mo177	11100	11568
Mo178	6189	6213
Mo181	8781	8807
Mo182	7815	8100
Mo186	6434	6791
Mo187	11500	11722
Mo188	5283	5333
Mo192	10200	9946
Mo194	5283	5346
Mo197	2359	2431
Mo198	7514	8962
Mo199	10300	11176
Mo200	8778	10066
Mo201	9488	10206
Mo202	4532	5349
Mo205	1412	2264
Mo206	6432	6738
Mo209	10300	10364
Mo210	6952	7282
Mo214	7812	8113
Mo218	7514	9858
Mo220	4712	4804
Mo222	6432	8188
Mo223	12000	11899

Mo224	4193	4374
Mo229	6186	6577
Mo230	7227	7561
Mo233	2529	2674
Mo236	5152	5190
Mo237	6252	6770
Mo240	7869	8006
Mo256	7570	7680
Mo258	6237	6369
Mo263	5771	5865
Mo264	7856	8508
Mo267	6627	7173
Mo268	1487	1594
Mo271	8896	9293
Mo272	9551	9946
Mo274	3355	3425
Mo275	5831	5909
Mo280	8896	9808
Mo282	5552	6843
Mo283	3259	4226
Mo286	3237	4569
Mo288	5290	5405
Mo292	10300	10788
Mo295	5797	5802
Mo296	12700	13559
Mo298	4966	5257
Mo300	7916	8678
Mo301	3649	4110
Mo303	9953	10891
Mo307	4971	5812
Mo309	5554	5670
Mo313	6516	6576
Mo318	6535	6645
Mo322	7734	8216
Mo326	4442	5166
Mo327	10600	10926
Mo329	11200	11327
Mo331	4799	4884
Mo332	10800	11839

Mo334	3208	3982
Mo337	9925	10129
Mo340	6451	7379
Mo344	5744	6484
Mo346	6039	6410
Mo349	7142	8755
Mo352	6917	7599
Mo353	5093	5312
Mo354	9594	10015
Mo355	6507	7240
Mo357	5265	5542
Mo358	5907	6000
Mo362	2452	2586
Mo364	15500	15881
Mo367	8290	8524
Mo368	6093	6100
Mo372	6599	6758
Mo373	3219	3294
Mo374	3708	3815
Mo379	8703	9682
Mo380	7764	8253
Mo381	4141	4213
Mo382	7100	7247
Mo383	5616	5859
Mo384	5014	5323

Table S2 Normalized cycle-threshold values and standard deviations (three biological replicates) for eight IBM RILs and eight diverse maize lines.

Target gene	Line	Cycle threshold	Standard deviation
PARP-LIKE	MO197	1.241668674	0.006786
PARP-LIKE	MO362	1.293685612	0.08108
PARP-LIKE	MO373	1.354176505	0.020955
PARP-LIKE	MO283	1.174018404	0.048811
PARP-LIKE	MO210	1.286510244	0.052169
PARP-LIKE	MO248	1.222963333	0.093895
PARP-LIKE	MO335	1.240960491	0.034316
PARP-LIKE	MO321	1.218637521	0.008838
PUTATIVE EST1	MO197	1.417610519	0.023636
PUTATIVE EST1	MO362	1.517526016	0.050229
PUTATIVE EST1	MO373	1.537128137	0.049222
PUTATIVE EST1	MO283	1.369568884	0.046174
PUTATIVE EST1	MO210	1.443887545	0.037885
PUTATIVE EST1	MO248	1.332528801	0.207698
PUTATIVE EST1	MO335	1.397600739	0.05074
PUTATIVE EST1	MO321	1.394557642	0.097539
HSP70-LIKE	MO197	1.467208451	0.009187
HSP70-LIKE	MO362	1.415032235	0.171624
HSP70-LIKE	MO373	1.583043951	0.041531
HSP70-LIKE	MO283	1.447171364	0.026302
HSP70-LIKE	MO210	1.342366751	0.09966
HSP70-LIKE	MO248	1.262786481	0.310692
HSP70-LIKE	MO335	1.350350859	0.208285
HSP70-LIKE	MO321	1.380526249	0.167299
PUTATIVE MCM	MO197	1.418322	0.072795
PUTATIVE MCM	MO362	1.403685	0.007432
PUTATIVE MCM	MO373	2.061854	0.058825
PUTATIVE MCM	MO283	1.534249	0.137605
PUTATIVE MCM	MO210	1.463066	0.023283
PUTATIVE MCM	MO248	1.350345	0.12441
PUTATIVE MCM	MO335	1.406862	0.261909
PUTATIVE MCM	MO321	1.429118	0.206596
SMC5-LIKE	MO197	1.567572	0.016133
SMC5-LIKE	MO362	1.493255	0.062939

SMC5-LIKE	MO373	1.859524	0.344072
SMC5-LIKE	MO283	1.454673	0.017631
SMC5-LIKE	MO210	1.637465	0.015299
SMC5-LIKE	MO248	1.3999	0.266683
SMC5-LIKE	MO335	1.357191	0.110716
SMC5-LIKE	MO321	1.60524	0.270395
XRCC3	MO197	1.646968	0.009864
XRCC3	MO362	1.482738	0.062059
XRCC3	MO373	1.744086	0.097184
XRCC3	MO283	1.545842	0.039877
XRCC3	MO210	1.703156	0.02457
XRCC3	MO248	1.282744	0.335574
XRCC3	MO335	1.505369	0.330892
XRCC3	MO321	1.475719	0.241425
RECQL	MO197	1.649675	0.031033
RECQL	MO362	1.782929	0.053057
RECQL	MO373	1.794913	0.019742
RECQL	MO283	1.689348	0.083935
RECQL	MO210	1.682722	0.01906
RECQL	MO248	1.489797	0.283075
RECQL	MO335	1.607421	0.162937
RECQL	MO321	1.565708	0.157492
PUTATIVE RFC	MO197	1.514247	0.042343
PUTATIVE RFC	MO362	1.705802	0.044018
PUTATIVE RFC	MO373	1.73969	0.040219
PUTATIVE RFC	MO283	1.612131	0.087136
PUTATIVE RFC	MO210	1.635361	0.035278
PUTATIVE RFC	MO248	1.443915	0.286868
PUTATIVE RFC	MO335	1.62957	0.17709
PUTATIVE RFC	MO321	1.617967	0.137971
IBP2	MO197	1.432537	0.037652
IBP2	MO362	1.591832	0.060282
IBP2	MO373	1.015068	0.02112
IBP2	MO283	1.545268	0.020074
IBP2	MO210	1.510611	0.014906
IBP2	MO248	1.420354	0.22317
IBP2	MO335	1.491292	0.179819
IBP2	MO321	1.499502	0.008311
SMH3	MO197	1.519152	0.023879

SMH3	MO362	1.602232	0.063258
SMH3	MO373	1.601164	0.070242
SMH3	MO283	1.50657	0.024483
SMH3	MO210	1.670968	0.161941
SMH3	MO248	1.385629	0.160458
SMH3	MO335	1.497588	0.188862
SMH3	MO321	1.515289	0.138896
SMH4	MO197	1.623037	0.027203
SMH4	MO362	1.772665	0.057581
SMH4	MO373	1.744536	0.062981
SMH4	MO283	1.633563	0.052874
SMH4	MO210	1.718631	0.009067
SMH4	MO248	1.524996	0.197052
SMH4	MO335	1.572396	0.131121
SMH4	MO321	1.674034	0.136519
SMH6	MO197	1.415475	0.031467
SMH6	MO362	1.531329	0.052214
SMH6	MO373	1.535289	0.0092
SMH6	MO283	1.416727	0.038031
SMH6	MO210	1.504578	0.02169
SMH6	MO248	1.296824	0.160051
SMH6	MO335	1.410144	0.100822
SMH6	MO321	1.331276	0.202895
TERT	MO197	1.836032	0.015616
TERT	MO362	1.802784	0.160938
TERT	MO373	1.843444	0.066245
TERT	MO283	1.562567	0.379365
TERT	MO210	1.754279	0.087318
TERT	MO248	1.961185	0.097632
TERT	MO335	1.544037	0.439238
TERT	MO321	1.731098	0.163184
KU70	MO197	1.66952	0.020245
KU70	MO362	1.731122	0.083006
KU70	MO373	1.711978	0.029323
KU70	MO283	1.747221	0.200764
KU70	MO210	1.654062	0.054408
KU70	MO248	1.818524	0.101623
KU70	MO335	1.599207	0.154499
KU70	MO321	1.694916	0.077016

KU80	MO197	1.5773	0.031897
KU80	MO362	1.692818	0.056767
KU80	MO373	1.634057	0.034175
KU80	MO283	1.513787	0.299636
KU80	MO210	1.510744	0.045729
KU80	MO248	1.622585	0.072229
KU80	MO335	1.619722	0.0388
KU80	MO321	1.640308	0.070783
PARP-LIKE	MO18W	1.237664	0.042466
PARP-LIKE	KI11	1.210849	0.011961
PARP-LIKE	B73	1.235157	0.022098
PARP-LIKE	NC358	1.219922	0.069116
PARP-LIKE	MO17	1.262547	0.014363
PARP-LIKE	OH43	1.240475	0.017633
PARP-LIKE	IL14H	1.213394	0.044747
PARP-LIKE	M37W	1.25984	0.03575
PUTATIVE EST1	MO18W	1.303916	0.217252
PUTATIVE EST1	KI11	1.380412	0.02618
PUTATIVE EST1	B73	1.439198	0.024551
PUTATIVE EST1	NC358	1.355281	0.101339
PUTATIVE EST1	MO17	1.368602	0.057013
PUTATIVE EST1	OH43	1.314027	0.149082
PUTATIVE EST1	IL14H	1.36223	0.056793
PUTATIVE EST1	M37W	1.386288	0.069043
HSP70-LIKE	MO18W	1.426597	0.168564
HSP70-LIKE	KI11	1.433594	0.078997
HSP70-LIKE	B73	1.429049	0.094366
HSP70-LIKE	NC358	1.548165	0.175916
HSP70-LIKE	MO17	1.439964	0.114499
HSP70-LIKE	OH43	1.527818	0.190242
HSP70-LIKE	IL14H	1.413337	0.116967
HSP70-LIKE	M37W	1.454468	0.101441
PUTATIVE MCM	MO18W	1.300044	0.138587
PUTATIVE MCM	KI11	1.255057	0.044643
PUTATIVE MCM	B73	1.299489	0.071733
PUTATIVE MCM	NC358	1.243755	0.081508
PUTATIVE MCM	MO17	1.36957	0.157688
PUTATIVE MCM	OH43	1.24588	0.034787

PUTATIVE MCM	IL14H	1.240513	0.086673
PUTATIVE MCM	M37W	1.416294	0.19479
SMC5-LIKE	MO18W	1.389646	0.193741
SMC5-LIKE	KI11	1.402251	0.055758
SMC5-LIKE	B73	1.463071	0.091063
SMC5-LIKE	NC358	1.395457	0.108746
SMC5-LIKE	MO17	1.480332	0.091424
SMC5-LIKE	OH43	1.385101	0.1001
SMC5-LIKE	IL14H	1.441629	0.069991
SMC5-LIKE	M37W	1.419765	0.082305
XRCC3	MO18W	1.528432	0.129868
XRCC3	KI11	1.560306	0.013758
XRCC3	B73	1.495064	0.041087
XRCC3	NC358	1.598055	0.051169
XRCC3	MO17	1.495625	0.093299
XRCC3	OH43	1.453364	0.151196
XRCC3	IL14H	1.497271	0.082068
XRCC3	M37W	1.462754	0.083486
RECQL	MO18W	1.640461	0.007362
RECQL	KI11	1.571467	0.047166
RECQL	B73	1.574081	0.155095
RECQL	NC358	1.580972	0.049788
RECQL	MO17	1.706311	0.214039
RECQL	OH43	1.485017	0.127722
RECQL	IL14H	1.508069	0.195518
RECQL	M37W	1.534521	0.157792
PUTATIVE RFC	MO18W	1.60569	0.035679
PUTATIVE RFC	KI11	1.474252	0.052909
PUTATIVE RFC	B73	1.560109	0.07837
PUTATIVE RFC	NC358	1.536142	0.057657
PUTATIVE RFC	MO17	1.626125	0.082774
PUTATIVE RFC	OH43	1.525453	0.039853
PUTATIVE RFC	IL14H	1.491629	0.072301
PUTATIVE RFC	M37W	1.504513	0.115874
IBP2	MO18W	1.434553	0.222445
IBP2	KI11	1.445433	0.059597
IBP2	B73	1.410179	0.030365
IBP2	NC358	1.553712	0.094549
IBP2	MO17	1.592566	0.146177

IBP2	OH43	1.380345	0.09466
IBP2	IL14H	1.434657	0.034863
IBP2	M37W	1.475062	0.111004
SMH3	MO18W	1.482783	0.16801
SMH3	KI11	1.519951	0.076638
SMH3	B73	1.528175	0.10172
SMH3	NC358	1.502994	0.113531
SMH3	MO17	1.512695	0.155886
SMH3	OH43	1.487138	0.21195
SMH3	IL14H	1.489314	0.098667
SMH3	M37W	1.488751	0.145295
SMH4	MO18W	1.679474	0.00876
SMH4	KI11	1.597676	0.061331
SMH4	B73	1.592792	0.064849
SMH4	NC358	1.586598	0.07472
SMH4	MO17	1.595241	0.080801
SMH4	OH43	1.596146	0.047868
SMH4	IL14H	1.601407	0.036304
SMH4	M37W	1.621854	0.082892
SMH6	MO18W	1.387577	0.235644
SMH6	KI11	1.45339	0.115297
SMH6	B73	1.352425	0.208931
SMH6	NC358	1.432	0.172092
SMH6	MO17	1.36473	0.208296
SMH6	OH43	1.339229	0.277432
SMH6	IL14H	1.411804	0.158771
SMH6	M37W	1.401654	0.187704
TERT	MO18W	1.622031	0.431112
TERT	KI11	1.640726	0.17672
TERT	B73	1.646155	0.385219
TERT	NC358	1.629555	0.267457
TERT	MO17	1.774726	0.446229
TERT	OH43	1.52359	0.437409
TERT	IL14H	1.630652	0.261126
TERT	M37W	1.663361	0.376116
KU70	MO18W	1.711944	0.139723
KU70	KI11	1.66153	0.065249
KU70	B73	1.831944	0.155167
KU70	NC358	1.637044	0.038117

KU70	MO17	1.756718	0.112222
KU70	OH43	1.587461	0.180733
KU70	IL14H	1.71857	0.043897
KU70	M37W	1.751657	0.152787
KU80	MO18W	1.538729	0.231572
KU80	KI11	1.566441	0.063048
KU80	B73	1.615243	0.130061
KU80	NC358	1.522701	0.107205
KU80	MO17	1.578032	0.126897
KU80	OH43	1.524656	0.173058
KU80	IL14H	1.506829	0.088078
KU80	M37W	1.560323	0.115263

Table S3 Primers for qPCR analysis.

Target Gene	Gene ID [a]	Primer Name	Oligo Sequence (5'-3')
Tert	GRMZM2G167338	ZmTERT-F1	TTGGATTCAAGGGATGCTGC
		ZmTERT-R1	TGATGAGCTTATTCATTAGTTTAGGCA
Ibp2	GRMZM2G110309	ZmIBP2-F1	CTCGTCAGAGTTGGAGGCGT
		ZmIBP2-R1	GTTGCGAGAAGGTCGAATGC
Gapdh	GRMZM2G046804	ZmGAPDH-F1	CCTTGCTCCCCTTGCTAAGG
		ZmGAPDH-R1	TGCCACCTCTCCAGTCCTTG
Smh3	GRMZM2G023667	ZmSMH3-R1	AGCCTATGGTCTTTGACGCCTT
Smh4	GRMZM2G108424	ZmSMH4-R1	ACACCTTCGATGGCTTTGATGG
Smh3/4		ZmSMH34-F1	TACGGCGACTTGCAGACTCAAA
Ku80	GRMZM2G137968	ZmKU80-F1	TCGGTTCTTCCCCTAGCTCTG
		ZmKU80-R1	CACTCCATGCATCGAAGGCC
Ku70	GRMZM2G414496	ZmKU70-F1	TACAGGACTCAGCCGGTGTTTA
		ZmKU70-R1	CGCCTGAGCAACCCAAAGAG
Smh6	GRMZM2G095239	ZmSMH6-F1	GCGCTTTTGACTGGTCGGGT
		ZmSMH56-R1	ATTCCAGCTCTAAGAGCAGCCT
Putative Est1	GRMZM2G018775	ZmEST-F2	GCAACGAGGTGGTGGCTGTT
		ZmEST-R2	GGCCGTGGGGGTAATGTCTT
Rad51L	GRMZM2G157817	ZmXRCC3-F1	TCACCAAGCTCTCACTCGGC
		ZmXRCC3-R1	AGGAGGGCGAGCTGAAGACA
Putative Rpa32	GRMZM2G061485	ZmRpA-F1	TGTTTGGCTTCTGGGGAGGG
		ZmRpA-R1	CAGTGATCCACCGCATGAGC
Smc5-like	GRMZM2G440916	ZmSmc5/6-F1	CACTCTGGATGCTTCGGACC
		ZmSmc5/6-R1	AATGGCCGTCTCCTTGTGCC
Parp-like	GRMZM2G145236	ZmPARP-F1	GGCACTCTCCTTACACCAGC
		ZmPARP-R1	GGCACTGCTTAGATCCAGGG

RecQL	GRMZM2G001160	ZmRecQ-F1	TCTACGGGGAGGATGGTCTG
		ZmRecQ-R1	GTTGCCACATGCCTCGGTCT
Hsp70-	GRMZM2G106429	ZmHsp70-F1	GGACTGCCTCAGCGATGCTA
like		ZmHsp70-R1	CCTCGTCAGGGTTGATGCTC
Putative	GRMZM2G112074	ZmMcm7-F1	TTCCAGCATCCGCCAAGAGG
Mcm		ZmMcm7-R1	ATCAGCCGCAGTGCTTCGTC
Putative	GRMZM2G457381	ZmRfC-F1	TGAAGCCGCAGGACAAGAGC
Rfc		ZmRfC-R1	TGGTCACAGGACGCCAATGC

[a]Gene model ID from http://maizesequence.org.
