# Supplementary material for: QTL Mapping and Candidate Gene Analysis of Telomere Length Control Factors in Maize (Zea mays L.)
Source: G3 (Bethesda). 2011 Nov 1;1(6):437–50. doi: 10.1534/g3.111.000703 (PMC3276162; doi:10.1534/g3.111.000703)
Supplement: Supporting Information [file supp_1.6.437_FigureS1.pdf]

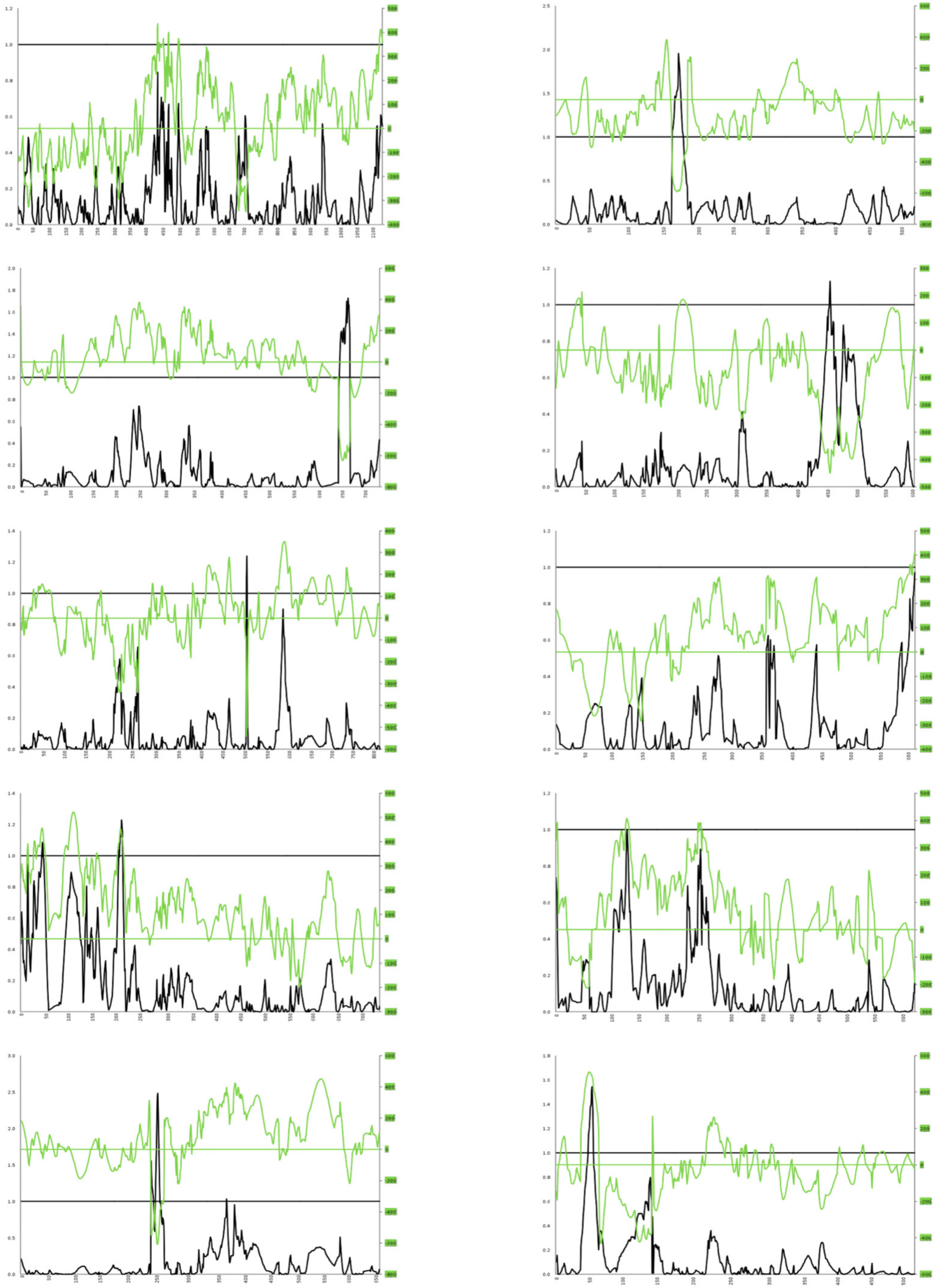

Figure S1A Black lines represent the QTL Likelihood for TEL_MD and the comparison-wise significance threshold (α = 0.01) at y=1 (left axis). Green lines represent the additive effect estimate and the boundary for changes in direction of effects at y=o (right green axis).

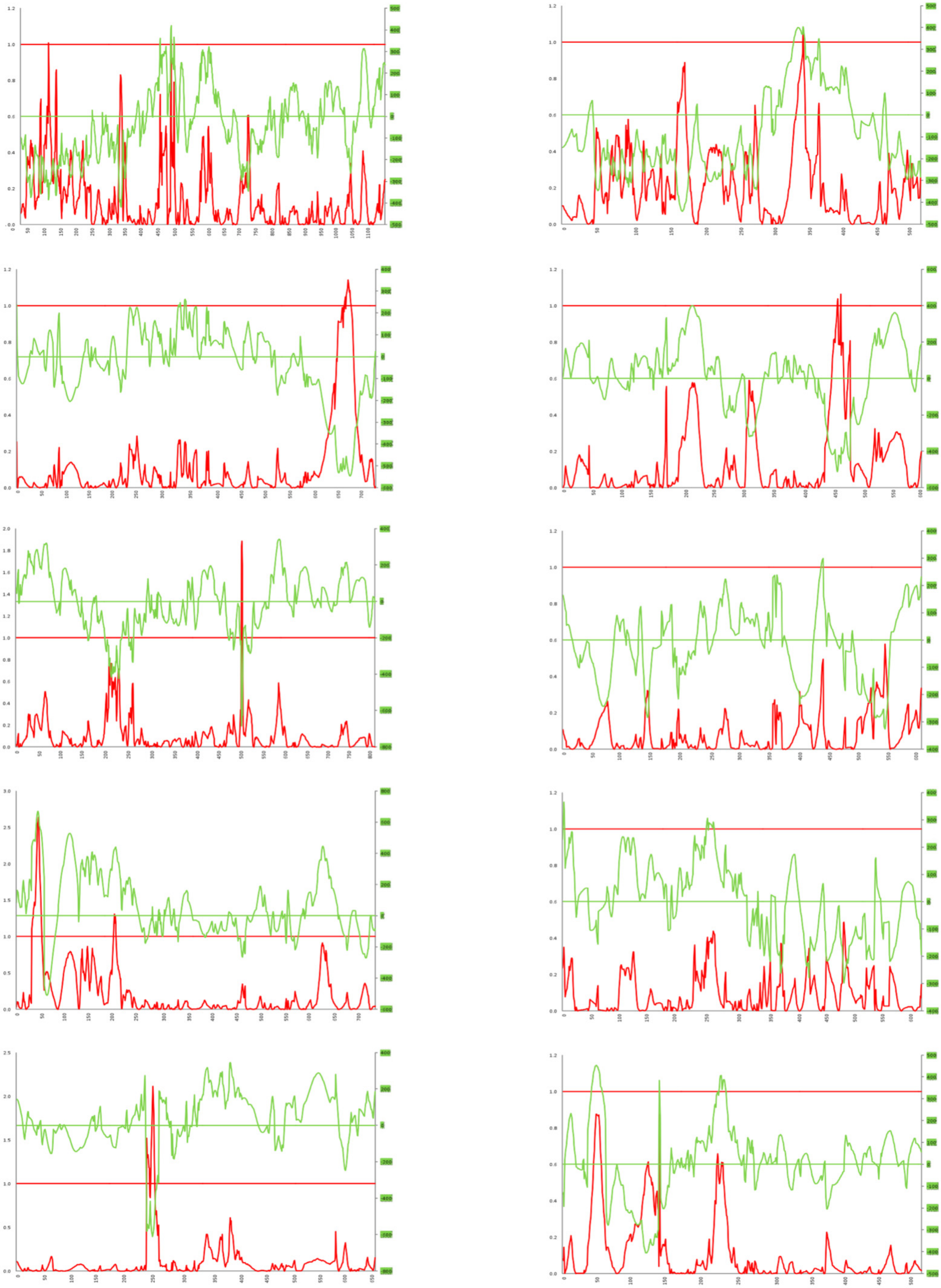

Figure S1B Red lines represent the QTL Likelihood for TEL_MN and the comparison-wise significance threshold (α = 0.01) at y=1 (left axis). Green lines represent the additive effect estimate and the boundary for changes in direction of effects at y=o (right green axis).
