# Supplementary material for: QTL Mapping and Candidate Gene Analysis of Telomere Length Control Factors in Maize (Zea mays L.)
Source: G3 (Bethesda). 2011 Nov 1;1(6):437–50. doi: 10.1534/g3.111.000703 (PMC3276162; doi:10.1534/g3.111.000703)
Supplement: Supporting Information [file supp_1.6.437_TableS1.pdf]

Table S1 Telomere lengths for maize intermated B73 × Mo17 (IBM) recombinant inbred lines used for quantitative-trait-locus analysis.

Line	TEL-MD	TEL-MN
Mo001	7266	7502
Mo003	5566	6385
Mo005	5874	5927
Mo007	7627	7314
Mo011	6490	6577
Mo012	5595	6089
Mo013	6011	6215
Mo014	6839	6424
Mo015	6545	6468
Mo016	7180	7984
Mo017	5829	6521
Mo018	6707	7001
Mo019	3649	4225
Mo021	4928	5173
Mo022	4505	4556
Mo023	7473	7765
Mo024	4164	4189
Mo025	6705	6842
Mo026	6246	6354
Mo027	6566	6693
Mo028	8413	9126
Mo029	3951	4364
Mo030	6395	6690
Mo034	6171	8928
Mo038	3976	4572
Mo039	4575	4905
Mo040	11400	11802
Mo041	5610	5777
Mo042	5836	5896
Mo043	6501	7580
Mo044	8209	8906
Mo046	4897	4902
Mo047	4814	4860
Mo051	3937	4477
Mo052	5201	5330
Mo054	5117	5187

Mo055	3530	3668
Mo057	3742	3954
Mo058	6876	7269
Mo060	7741	8054
Mo061	3685	3816
Mo063	3555	3772
Mo067	5375	5555
Mo068	6274	7177
Mo071	7897	8738
Mo074	2770	2967
Mo080	5948	5977
Mo081	5503	5939
Mo083	5503	6561
Mo085	4264	4551
Mo092	2958	4136
Mo093	4905	5396
Mo096	7822	7860
Mo097	4915	4928
Mo106	5547	5531
Mo109	3332	3251
Mo111	1667	2437
Mo113	5443	5502
Mo114	5713	5759
Mo116	5713	5919
Mo118	5237	5653
Mo119	3555	3772
Mo120	7563	7916
Mo124	3951	4023
Mo127	3420	3607
Mo128	7047	7264
Mo130	4587	4930
Mo132	4756	4985
Mo134	3057	3140
Mo138	7123	7709
Mo141	4930	5626
Mo142	4755	5678
Mo143	5693	6429
Mo145	5298	5472
Mo146	4587	4676

Mo147	2968	3076
Mo150	8633	8477
Mo151	1956	3021
Mo153	4491	4467
Mo154	6168	6182
Mo156	6659	6975
Mo157	5713	5796
Mo159	3749	4037
Mo160	3343	4348
Mo164	4048	4213
Mo167	9513	9619
Mo168	6452	6871
Mo172	7536	7373
Mo174	7834	7980
Mo176	7249	7232
Mo177	11100	11568
Mo178	6189	6213
Mo181	8781	8807
Mo182	7815	8100
Mo186	6434	6791
Mo187	11500	11722
Mo188	5283	5333
Mo192	10200	9946
Mo194	5283	5346
Mo197	2359	2431
Mo198	7514	8962
Mo199	10300	11176
Mo200	8778	10066
Mo201	9488	10206
Mo202	4532	5349
Mo205	1412	2264
Mo206	6432	6738
Mo209	10300	10364
Mo210	6952	7282
Mo214	7812	8113
Mo218	7514	9858
Mo220	4712	4804
Mo222	6432	8188
Mo223	12000	11899

Mo224	4193	4374
Mo229	6186	6577
Mo230	7227	7561
Mo233	2529	2674
Mo236	5152	5190
Mo237	6252	6770
Mo240	7869	8006
Mo256	7570	7680
Mo258	6237	6369
Mo263	5771	5865
Mo264	7856	8508
Mo267	6627	7173
Mo268	1487	1594
Mo271	8896	9293
Mo272	9551	9946
Mo274	3355	3425
Mo275	5831	5909
Mo280	8896	9808
Mo282	5552	6843
Mo283	3259	4226
Mo286	3237	4569
Mo288	5290	5405
Mo292	10300	10788
Mo295	5797	5802
Mo296	12700	13559
Mo298	4966	5257
Mo300	7916	8678
Mo301	3649	4110
Mo303	9953	10891
Mo307	4971	5812
Mo309	5554	5670
Mo313	6516	6576
Mo318	6535	6645
Mo322	7734	8216
Mo326	4442	5166
Mo327	10600	10926
Mo329	11200	11327
Mo331	4799	4884
Mo332	10800	11839

Mo334	3208	3982
Mo337	9925	10129
Mo340	6451	7379
Mo344	5744	6484
Mo346	6039	6410
Mo349	7142	8755
Mo352	6917	7599
Mo353	5093	5312
Mo354	9594	10015
Mo355	6507	7240
Mo357	5265	5542
Mo358	5907	6000
Mo362	2452	2586
Mo364	15500	15881
Mo367	8290	8524
Mo368	6093	6100
Mo372	6599	6758
Mo373	3219	3294
Mo374	3708	3815
Mo379	8703	9682
Mo380	7764	8253
Mo381	4141	4213
Mo382	7100	7247
Mo383	5616	5859
Mo384	5014	5323
