# Supplementary material for: QTL Mapping and Candidate Gene Analysis of Telomere Length Control Factors in Maize (Zea mays L.)
Source: G3 (Bethesda). 2011 Nov 1;1(6):437–50. doi: 10.1534/g3.111.000703 (PMC3276162; doi:10.1534/g3.111.000703)
Supplement: Supporting Information [file supp_1.6.437_TableS2.pdf]

Table S2 Normalized cycle-threshold values and standard deviations (three biological replicates) for eight IBM RILs and eight diverse maize lines.

Target gene	Line	Cycle threshold	Standard deviation
PARP-LIKE	MO197	1.241668674	0.006786
PARP-LIKE	MO362	1.293685612	0.08108
PARP-LIKE	MO373	1.354176505	0.020955
PARP-LIKE	MO283	1.174018404	0.048811
PARP-LIKE	MO210	1.286510244	0.052169
PARP-LIKE	MO248	1.222963333	0.093895
PARP-LIKE	MO335	1.240960491	0.034316
PARP-LIKE	MO321	1.218637521	0.008838
PUTATIVE EST1	MO197	1.417610519	0.023636
PUTATIVE EST1	MO362	1.517526016	0.050229
PUTATIVE EST1	MO373	1.537128137	0.049222
PUTATIVE EST1	MO283	1.369568884	0.046174
PUTATIVE EST1	MO210	1.443887545	0.037885
PUTATIVE EST1	MO248	1.332528801	0.207698
PUTATIVE EST1	MO335	1.397600739	0.05074
PUTATIVE EST1	MO321	1.394557642	0.097539
HSP70-LIKE	MO197	1.467208451	0.009187
HSP70-LIKE	MO362	1.415032235	0.171624
HSP70-LIKE	MO373	1.583043951	0.041531
HSP70-LIKE	MO283	1.447171364	0.026302
HSP70-LIKE	MO210	1.342366751	0.09966
HSP70-LIKE	MO248	1.262786481	0.310692
HSP70-LIKE	MO335	1.350350859	0.208285
HSP70-LIKE	MO321	1.380526249	0.167299
PUTATIVE MCM	MO197	1.418322	0.072795
PUTATIVE MCM	MO362	1.403685	0.007432
PUTATIVE MCM	MO373	2.061854	0.058825
PUTATIVE MCM	MO283	1.534249	0.137605
PUTATIVE MCM	MO210	1.463066	0.023283
PUTATIVE MCM	MO248	1.350345	0.12441
PUTATIVE MCM	MO335	1.406862	0.261909
PUTATIVE MCM	MO321	1.429118	0.206596
SMC5-LIKE	MO197	1.567572	0.016133
SMC5-LIKE	MO362	1.493255	0.062939

SMC5-LIKE	MO373	1.859524	0.344072
SMC5-LIKE	MO283	1.454673	0.017631
SMC5-LIKE	MO210	1.637465	0.015299
SMC5-LIKE	MO248	1.3999	0.266683
SMC5-LIKE	MO335	1.357191	0.110716
SMC5-LIKE	MO321	1.60524	0.270395
XRCC3	MO197	1.646968	0.009864
XRCC3	MO362	1.482738	0.062059
XRCC3	MO373	1.744086	0.097184
XRCC3	MO283	1.545842	0.039877
XRCC3	MO210	1.703156	0.02457
XRCC3	MO248	1.282744	0.335574
XRCC3	MO335	1.505369	0.330892
XRCC3	MO321	1.475719	0.241425
RECQL	MO197	1.649675	0.031033
RECQL	MO362	1.782929	0.053057
RECQL	MO373	1.794913	0.019742
RECQL	MO283	1.689348	0.083935
RECQL	MO210	1.682722	0.01906
RECQL	MO248	1.489797	0.283075
RECQL	MO335	1.607421	0.162937
RECQL	MO321	1.565708	0.157492
PUTATIVE RFC	MO197	1.514247	0.042343
PUTATIVE RFC	MO362	1.705802	0.044018
PUTATIVE RFC	MO373	1.73969	0.040219
PUTATIVE RFC	MO283	1.612131	0.087136
PUTATIVE RFC	MO210	1.635361	0.035278
PUTATIVE RFC	MO248	1.443915	0.286868
PUTATIVE RFC	MO335	1.62957	0.17709
PUTATIVE RFC	MO321	1.617967	0.137971
IBP2	MO197	1.432537	0.037652
IBP2	MO362	1.591832	0.060282
IBP2	MO373	1.015068	0.02112
IBP2	MO283	1.545268	0.020074
IBP2	MO210	1.510611	0.014906
IBP2	MO248	1.420354	0.22317
IBP2	MO335	1.491292	0.179819
IBP2	MO321	1.499502	0.008311
SMH3	MO197	1.519152	0.023879

SMH3	MO362	1.602232	0.063258
SMH3	MO373	1.601164	0.070242
SMH3	MO283	1.50657	0.024483
SMH3	MO210	1.670968	0.161941
SMH3	MO248	1.385629	0.160458
SMH3	MO335	1.497588	0.188862
SMH3	MO321	1.515289	0.138896
SMH4	MO197	1.623037	0.027203
SMH4	MO362	1.772665	0.057581
SMH4	MO373	1.744536	0.062981
SMH4	MO283	1.633563	0.052874
SMH4	MO210	1.718631	0.009067
SMH4	MO248	1.524996	0.197052
SMH4	MO335	1.572396	0.131121
SMH4	MO321	1.674034	0.136519
SMH6	MO197	1.415475	0.031467
SMH6	MO362	1.531329	0.052214
SMH6	MO373	1.535289	0.0092
SMH6	MO283	1.416727	0.038031
SMH6	MO210	1.504578	0.02169
SMH6	MO248	1.296824	0.160051
SMH6	MO335	1.410144	0.100822
SMH6	MO321	1.331276	0.202895
TERT	MO197	1.836032	0.015616
TERT	MO362	1.802784	0.160938
TERT	MO373	1.843444	0.066245
TERT	MO283	1.562567	0.379365
TERT	MO210	1.754279	0.087318
TERT	MO248	1.961185	0.097632
TERT	MO335	1.544037	0.439238
TERT	MO321	1.731098	0.163184
KU70	MO197	1.66952	0.020245
KU70	MO362	1.731122	0.083006
KU70	MO373	1.711978	0.029323
KU70	MO283	1.747221	0.200764
KU70	MO210	1.654062	0.054408
KU70	MO248	1.818524	0.101623
KU70	MO335	1.599207	0.154499
KU70	MO321	1.694916	0.077016

KU80	MO197	1.5773	0.031897
KU80	MO362	1.692818	0.056767
KU80	MO373	1.634057	0.034175
KU80	MO283	1.513787	0.299636
KU80	MO210	1.510744	0.045729
KU80	MO248	1.622585	0.072229
KU80	MO335	1.619722	0.0388
KU80	MO321	1.640308	0.070783
PARP-LIKE	MO18W	1.237664	0.042466
PARP-LIKE	KI11	1.210849	0.011961
PARP-LIKE	B73	1.235157	0.022098
PARP-LIKE	NC358	1.219922	0.069116
PARP-LIKE	MO17	1.262547	0.014363
PARP-LIKE	OH43	1.240475	0.017633
PARP-LIKE	IL14H	1.213394	0.044747
PARP-LIKE	M37W	1.25984	0.03575
PUTATIVE EST1	MO18W	1.303916	0.217252
PUTATIVE EST1	KI11	1.380412	0.02618
PUTATIVE EST1	B73	1.439198	0.024551
PUTATIVE EST1	NC358	1.355281	0.101339
PUTATIVE EST1	MO17	1.368602	0.057013
PUTATIVE EST1	OH43	1.314027	0.149082
PUTATIVE EST1	IL14H	1.36223	0.056793
PUTATIVE EST1	M37W	1.386288	0.069043
HSP70-LIKE	MO18W	1.426597	0.168564
HSP70-LIKE	KI11	1.433594	0.078997
HSP70-LIKE	B73	1.429049	0.094366
HSP70-LIKE	NC358	1.548165	0.175916
HSP70-LIKE	MO17	1.439964	0.114499
HSP70-LIKE	OH43	1.527818	0.190242
HSP70-LIKE	IL14H	1.413337	0.116967
HSP70-LIKE	M37W	1.454468	0.101441
PUTATIVE MCM	MO18W	1.300044	0.138587
PUTATIVE MCM	KI11	1.255057	0.044643
PUTATIVE MCM	B73	1.299489	0.071733
PUTATIVE MCM	NC358	1.243755	0.081508
PUTATIVE MCM	MO17	1.36957	0.157688
PUTATIVE MCM	OH43	1.24588	0.034787

PUTATIVE MCM	IL14H	1.240513	0.086673
PUTATIVE MCM	M37W	1.416294	0.19479
SMC5-LIKE	MO18W	1.389646	0.193741
SMC5-LIKE	KI11	1.402251	0.055758
SMC5-LIKE	B73	1.463071	0.091063
SMC5-LIKE	NC358	1.395457	0.108746
SMC5-LIKE	MO17	1.480332	0.091424
SMC5-LIKE	OH43	1.385101	0.1001
SMC5-LIKE	IL14H	1.441629	0.069991
SMC5-LIKE	M37W	1.419765	0.082305
XRCC3	MO18W	1.528432	0.129868
XRCC3	KI11	1.560306	0.013758
XRCC3	B73	1.495064	0.041087
XRCC3	NC358	1.598055	0.051169
XRCC3	MO17	1.495625	0.093299
XRCC3	OH43	1.453364	0.151196
XRCC3	IL14H	1.497271	0.082068
XRCC3	M37W	1.462754	0.083486
RECQL	MO18W	1.640461	0.007362
RECQL	KI11	1.571467	0.047166
RECQL	B73	1.574081	0.155095
RECQL	NC358	1.580972	0.049788
RECQL	MO17	1.706311	0.214039
RECQL	OH43	1.485017	0.127722
RECQL	IL14H	1.508069	0.195518
RECQL	M37W	1.534521	0.157792
PUTATIVE RFC	MO18W	1.60569	0.035679
PUTATIVE RFC	KI11	1.474252	0.052909
PUTATIVE RFC	B73	1.560109	0.07837
PUTATIVE RFC	NC358	1.536142	0.057657
PUTATIVE RFC	MO17	1.626125	0.082774
PUTATIVE RFC	OH43	1.525453	0.039853
PUTATIVE RFC	IL14H	1.491629	0.072301
PUTATIVE RFC	M37W	1.504513	0.115874
IBP2	MO18W	1.434553	0.222445
IBP2	KI11	1.445433	0.059597
IBP2	B73	1.410179	0.030365
IBP2	NC358	1.553712	0.094549
IBP2	MO17	1.592566	0.146177

IBP2	OH43	1.380345	0.09466
IBP2	IL14H	1.434657	0.034863
IBP2	M37W	1.475062	0.111004
SMH3	MO18W	1.482783	0.16801
SMH3	KI11	1.519951	0.076638
SMH3	B73	1.528175	0.10172
SMH3	NC358	1.502994	0.113531
SMH3	MO17	1.512695	0.155886
SMH3	OH43	1.487138	0.21195
SMH3	IL14H	1.489314	0.098667
SMH3	M37W	1.488751	0.145295
SMH4	MO18W	1.679474	0.00876
SMH4	KI11	1.597676	0.061331
SMH4	B73	1.592792	0.064849
SMH4	NC358	1.586598	0.07472
SMH4	MO17	1.595241	0.080801
SMH4	OH43	1.596146	0.047868
SMH4	IL14H	1.601407	0.036304
SMH4	M37W	1.621854	0.082892
SMH6	MO18W	1.387577	0.235644
SMH6	KI11	1.45339	0.115297
SMH6	B73	1.352425	0.208931
SMH6	NC358	1.432	0.172092
SMH6	MO17	1.36473	0.208296
SMH6	OH43	1.339229	0.277432
SMH6	IL14H	1.411804	0.158771
SMH6	M37W	1.401654	0.187704
TERT	MO18W	1.622031	0.431112
TERT	KI11	1.640726	0.17672
TERT	B73	1.646155	0.385219
TERT	NC358	1.629555	0.267457
TERT	MO17	1.774726	0.446229
TERT	OH43	1.52359	0.437409
TERT	IL14H	1.630652	0.261126
TERT	M37W	1.663361	0.376116
KU70	MO18W	1.711944	0.139723
KU70	KI11	1.66153	0.065249
KU70	B73	1.831944	0.155167
KU70	NC358	1.637044	0.038117

KU70	MO17	1.756718	0.112222
KU70	OH43	1.587461	0.180733
KU70	IL14H	1.71857	0.043897
KU70	M37W	1.751657	0.152787
KU80	MO18W	1.538729	0.231572
KU80	KI11	1.566441	0.063048
KU80	B73	1.615243	0.130061
KU80	NC358	1.522701	0.107205
KU80	MO17	1.578032	0.126897
KU80	OH43	1.524656	0.173058
KU80	IL14H	1.506829	0.088078
KU80	M37W	1.560323	0.115263
