# Supplementary material for: QTL Mapping and Candidate Gene Analysis of Telomere Length Control Factors in Maize (Zea mays L.)
Source: G3 (Bethesda). 2011 Nov 1;1(6):437–50. doi: 10.1534/g3.111.000703 (PMC3276162; doi:10.1534/g3.111.000703)
Supplement: Supporting Information [file supp_1.6.437_TableS3.pdf]

Table S3 Primers for qPCR analysis.

Target Gene	Gene ID [a]	Primer Name	Oligo Sequence (5'-3')
Tert	GRMZM2G167338	ZmTERT-F1	TTGGATTCAAGGGATGCTGC
		ZmTERT-R1	TGATGAGCTTATTCATTAGTTTAGGCA
Ibp2	GRMZM2G110309	ZmIBP2-F1	CTCGTCAGAGTTGGAGGCGT
		ZmIBP2-R1	GTTGCGAGAAGGTCGAATGC
Gapdh	GRMZM2G046804	ZmGAPDH-F1	CCTTGCTCCCCTTGCTAAGG
		ZmGAPDH-R1	TGCCACCTCTCCAGTCCTTG
Smh3	GRMZM2G023667	ZmSMH3-R1	AGCCTATGGTCTTTGACGCCTT
Smh4	GRMZM2G108424	ZmSMH4-R1	ACACCTTCGATGGCTTTGATGG
Smh3/4		ZmSMH34-F1	TACGGCGACTTGCAGACTCAAA
Ku80	GRMZM2G137968	ZmKU80-F1	TCGGTTCTTCCCCTAGCTCTG
		ZmKU80-R1	CACTCCATGCATCGAAGGCC
Ku70	GRMZM2G414496	ZmKU70-F1	TACAGGACTCAGCCGGTGTTTA
		ZmKU70-R1	CGCCTGAGCAACCCAAAGAG
Smh6	GRMZM2G095239	ZmSMH6-F1	GCGCTTTTGACTGGTCGGGT
		ZmSMH56-R1	ATTCCAGCTCTAAGAGCAGCCT
Putative	GRMZM2G018775	ZmEST-F2	GCAACGAGGTGGTGGCTGTT
Est1		ZmEST-R2	GGCCGTGGGGGTAATGTCTT
Rad51L	GRMZM2G157817	ZmXRCC3-F1	TCACCAAGCTCTCACTCGGC
		ZmXRCC3-R1	AGGAGGGCGAGCTGAAGACA
Putative	GRMZM2G061485	ZmRpA-F1	TGTTTGGCTTCTGGGGAGGG
Rpa32		ZmRpA-R1	CAGTGATCCACCGCATGAGC
Smc5-like	GRMZM2G440916	ZmSmc5/6-F1	CACTCTGGATGCTTCGGACC
		ZmSmc5/6-R1	AATGGCCGTCTCCTTGTGCC
Parp-like	GRMZM2G145236	ZmPARP-F1	GGCACTCTCCTTACACCAGC
		ZmPARP-R1	GGCACTGCTTAGATCCAGGG

RecQL	GRMZM2G001160	ZmRecQ-F1	TCTACGGGGAGGATGGTCTG
		ZmRecQ-R1	GTTGCCACATGCCTCGGTCT
Hsp70-	GRMZM2G106429	ZmHsp70-F1	GGACTGCCTCAGCGATGCTA
like		ZmHsp70-R1	CCTCGTCAGGGTTGATGCTC
Putative	GRMZM2G112074	ZmMcm7-F1	TTCCAGCATCCGCCAAGAGG
Mcm		ZmMcm7-R1	ATCAGCCGCAGTGCTTCGTC
Putative	GRMZM2G457381	ZmRfC-F1	TGAAGCCGCAGGACAAGAGC
Rfc		ZmRfC-R1	TGGTCACAGGACGCCAATGC

[a]Gene model ID from http://maizesequence.org.
